# prm-PASEF-Based Quantification and Isomeric Model for Extended Coverage of Human Plasma Lipidome in Parkinson’s Disease

**DOI:** 10.1021/acs.analchem.5c02340

**Published:** 2025-10-27

**Authors:** Dhanwin Baker, Gabriel Gonzalez Escamilla, Daniel Janitschke, Yvan Devaux, Nils Schröter, Sergiu Groppa, Laura Bindila

**Affiliations:** a Clinical Lipidomics Unit, Institute of Physiological Chemistry, 39068 University Medical Center of the Johannes Gutenberg University Mainz, Duesbergweg 6, Mainz 55128, Germany; b Gabriel Gonzalez Escamilla (GGE) - Department of Neurology, Universitätsklinikum des Saarlandes, Kirrberger Straße 100, Homburg 66421, Germany; c Daniel Janitschke (DJ) - Department of Neurology, Universitätsklinikum des Saarlandes, Kirrberger Straße 100, Homburg 66421, Germany; d Yvan Devaux (YD) - Cardiovascular Research Unit, Department of Precision Health, Luxembourg Institute of Health, 1 A-B Rue Thomas Edison, Strassen 1445, Luxembourg; e Nils Schröter (NS) - Clinic for Neurology and Neurophysiology, Universität Klinikum Freiburg, Breisacher Straße 64, Freiburg 79106, Germany; f Sergiu Groppa (SG) - Department of Neurology, Universitätsklinikum des Saarlandes, Kirrberger Straße 100, Homburg 66421, Germany

## Abstract

This study introduces a clinical lipidomics platform leveraging fragment-based quantification on parallel reaction monitoring (PRM)-parallel accumulation serial fragmentation (PASEF) for lipid quantification. An isomeric model, termed “SN regression model”, built on specific PASEF-fragment ion patterns, was developed for the quantification of coeluting sn positional isomers without prior derivatization. This PASEF-isomeric lipidomics aids in the resolution and quantification of 176 lipid isomers coeluting in chromatography and/or ion mobility dimensions, expanding the lipidome quantitative coverage to 481 plasma lipids covering 14 lipid subclasses with CV <40% for 32 plasma replicates. We demonstrated the method’s advantage for clinical research by detailed quantitative lipidomic phenotyping of patients with Parkinson’s disease, enabling the delineation of new biochemical pathways affected by the disorder and stratification of patients. The method’s amenability for high-throughput deep quantitative coverage of highly structurally resolved lipidome has implications for improving the diagnosis and understanding of the distinct metabolic alterations in Parkinson’s disease subgroups and, generally, for disorders associated with lipid dysregulation.

## Graphical abstract

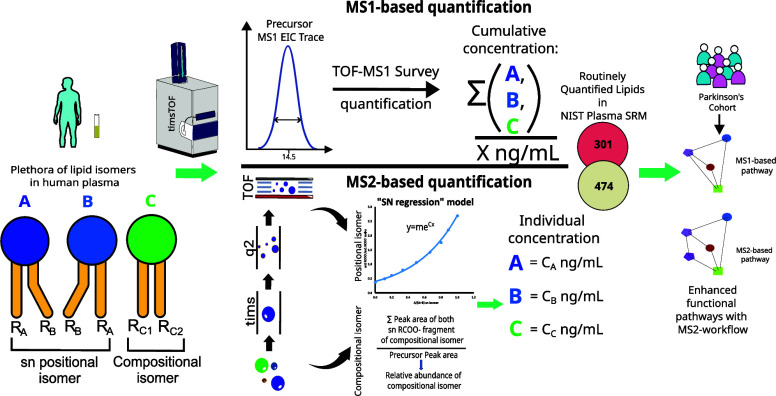

## Introduction

Lipidomics has made significant strides in recent years due to advancements in high-resolution mass spectrometry (HRMS) technologies^1,2^ pushing lipidomics into new territories.^3^ With HRMS, different acquisition modes, namely, Data-Dependent Acquisition (DDA) in MS2 to MS^*n*^ modes,^4^ Data-Independent Acquisition (DIA),^5^ Parallel Reaction Monitoring (PRM),^6^ or ion mobility (IM), offer unique advantages that can be combined for broad structural identifications of lipids in different applications.^7^

A major challenge in lipidomic analyses is the relative and/or absolute quantification of coeluting isomeric and isobaric lipids. The most functionally important isomeric forms for glycerophospholipids (PL) are (i) the sn positional isomers arising from the substitution of a fatty acyl (FA) on either sn1 or sn2 C atom of the glycerol backbone; (ii) compositional isomers, which differ in the types of FA chains attached to their sn1 and sn2 positions; and (iii) isomers differing in the double-bond (DB) position and/or the stereochemistry.^8^ Typically, coeluting isomers/isobars are quantified as a single cluster based on the MS1 precursor peak area. Coeluting isobaric lipids of different classes in complex matrices, such as human plasma, can be quantified using multiple reaction monitoring with specific class-based fragmentation patterns. Lipid derivatization^9–11^ strategies, such as most notably ozonolysis, have been developed to identify and quantify the DB-positional isomers. Such strategies introduce complexity in the matrix particularly when short gradients or direct infusion MS are used for very high-throughput profiling.^12^ SN positional isomers of lyso-glycerophospholipids can be readily resolved by reverse-phase liquid chromatography (RPLC).^13,14^ Yet most of the sn1, sn2 acyl positional and compositional isomers in doubly FA-substituted glycerophospholipids remain unresolved by RPLC.^15^ A shotgun MS^*n*^-based approach was reported by Ekroos et al.^16^ for the identification of such positional isomers using structure-specific fragment ions for relative quantification of the coeluting lipids. Glycerophospholipids are identified by their carboxylate fragment ions (sn RCOO^–^) derived from FA chains at the sn1 and sn2 positions as [M-H]^−^ and/or [M+HCOO]^−^ ions in negative ion mode.^17^ These fragments are observed in a fixed ratio relative to each other in an isomerically pure PLs.^18^ The abundance of the sn2 RCOO^–^ fragment typically surpasses that of the sn1 RCOO^–^ fragment,^19^ except in specific lipid classes like glycerophosphatidic acid (PA), glycerophosphoinositol (PI), and glycerophosphoserine (PS).^20^ Therefore, variations in the abundance ratio of these fragment ions can delineate the presence of SN positional isomers and thereby aid in their relative quantification as shown by Wu et al.,^21^ Zhao et al.,^22^ and Brugger et al..^23^

The integration of IM into HRMS, such as on trapped ion mobility (TIMS) can partially address the issue of coeluting isomers.^24,25^ It has been observed that the sn isomers of lysoglycerophospholipid species can be differentiated by ion mobility, and that the sn2 positional isomer has typically higher collisional cross-section (CCS) values than its sn1 counterpart,^14,26,27^ which can be used in general to confirm the annotation. However, ion mobility operating in routine resolution mode (*R*_p_ - 100) does not resolve sn positional isomers in doubly FA-substituted PLs. Koomen et al. and Xu et al.^28,29^ developed high-resolution demultiplexing (HRdm) of IM-MS to identify coeluting isomers in complex mixtures, highlighting the postprocessing capabilities to resolve sn position isomers (*R*_p_ - 250). However, the workflow is not yet implemented for quantification and utilizes the PNNL Preprocessor to demultiplex the ion mobility data, limiting its use for other vendors’ data formats. The expanding field of lipidomics calls for vendor-neutral workflows.^30^

Targeted methods like multiple reaction monitoring (MRM) and PRM enable quantification based on unique fragment ions of a lipid structure, thus distinguishing coeluting isomers.^31^ In addition, prm-Parallel Accumulation Serial Fragmentation (prm-PASEF)^32^ leverages targeted precursor selection based on retention time (RT), mass-to-charge ratio (*m*/*z*), collision energy, and ion mobility for increased selectivity and sensitivity. Such methods allow for monitoring of multiple precursor-fragment transitions from a single precursor ion, facilitating the individual isomer quantification.

In this study, we present a prm-PASEF-based targeted lipidomic profiling method enabling extended quantitative lipidome coverage in routine clinical profiling. We developed the workflow using a set of coeluting lipids determined in prior work^14,33^ to be relevant and robust for 4-dimensional (4D)-TIMS-based routine clinical profiling of human plasma. Characteristic fragment ions of coeluting isomers generated from their corresponding [M–H]^−^ and/or [M+HCOO]^−^ adduct ions were used for the identification and quantification. Using the fragment ion abundance obtained via prm-PASEF, we developed an “SN Regression” model for the quantification of sn positional isomers in PC, glycerophosphoethanolamine (PE), and PS lipid classes. This model was reasoned to account for variability in fragment ion intensity across different matrix effects of lipids, fragmentation efficiency of different species, MS^2^ spectral quality, and dynamic range of the biological mixtures and of the MS instrument. With prm-PASEF and “SN regression model”, we could routinely profile and quantify 481 lipids across 14 lipid classes, including 112 FA compositional isomers and 64 sn isomers in NIST Human Plasma SRM 1950 (NIST plasma SRM). We applied this method to a cohort of Parkinson’s disease (PD) patients and evidenced benefits for deep lipid quantitative coverage, PD-phenotyping, and in-depth pathway elucidation.

## Experimental Section

The serum samples from the Parkinson cohort with project number 837.311.12­(8412-F) used in this research complied with all relevant ethical regulations regarding the use of human study participants and were conducted following the criteria set by Rheinland-Pfalz, Germany.

### Sample Preparation

#### Liquid–Liquid Extraction for NIST Plasma SRM and Parkinson’s Disease Cohort Plasma

A liquid–liquid extraction (LLE) method using 800 μL MTBE/methanol (10:3; v/v) and 200 μL of 0.1% formic acid^14^ was used for the extraction of 20 μL NIST plasma SRM as previously described.^14^ The plasma samples were spiked with at least one internal standard (ISTD) per lipid class (Supplementary Table 1). The mixture was first vortexed at 1050 rpm and centrifuged at 5000 *g* to separate the two liquid phases. The upper organic phase was then separated, evaporated under a nitrogen stream, and stored at −20 °C. Before analysis, samples were reconstituted in 360 μL of 90% methanol.

This workflow was applied to a cohort of serum samples from 47 control individuals (HC) and 47 Parkinson’s disease (PD) patients (Supplementary Table 4). The cohort included 35 females and 59 males, ages ranging from 20 to 82 years old, to enable age and sex difference evaluations with PD.

### Reversed-Phase Liquid Chromatography TIMS PASEF

A μL-flow RPLC prm-PASEF workflow was devised to enable routine quantitative in-depth profiling of the plasma lipidome in clinical samples (Figure 1). The LC separation method consisted of a 20 min gradient at a flow rate of 0.2 mL/min using a C18 Luna Omega column (100 × 2.1 mm × 1.6 μm).^14^ Twenty and ten microliters were injected for negative and positive modes, respectively.^14^ The prm-PASEF experiments were performed in negative and positive polarities using TIMS-TOF instruments (Bruker Daltonics, Germany). The basic MS parameters used were: end plate offset of 500 V, capillary voltages of 4500 and 3600 V for negative and positive mode, respectively. Scan mode was set to PASEF with the mass scan range of 100–1350 Da for both MS and MS2 acquisition. The acquisition cycle consisted of 0.1 s with the mobility scan range of 0.55–1.87 V.s/cm^2^ for the positive mode and 0.55–1.86 V.s/cm^2^ for the negative mode.^14^ Other MS parameters were set as follows: MS interval setting −10 s. The mass isolation width −1 Da, the RT range for targets −60 s, and the mobility window −0.03 V.s/cm^2^. A target list template has been shown in Supplementary Data 1.

**Figure 1:**
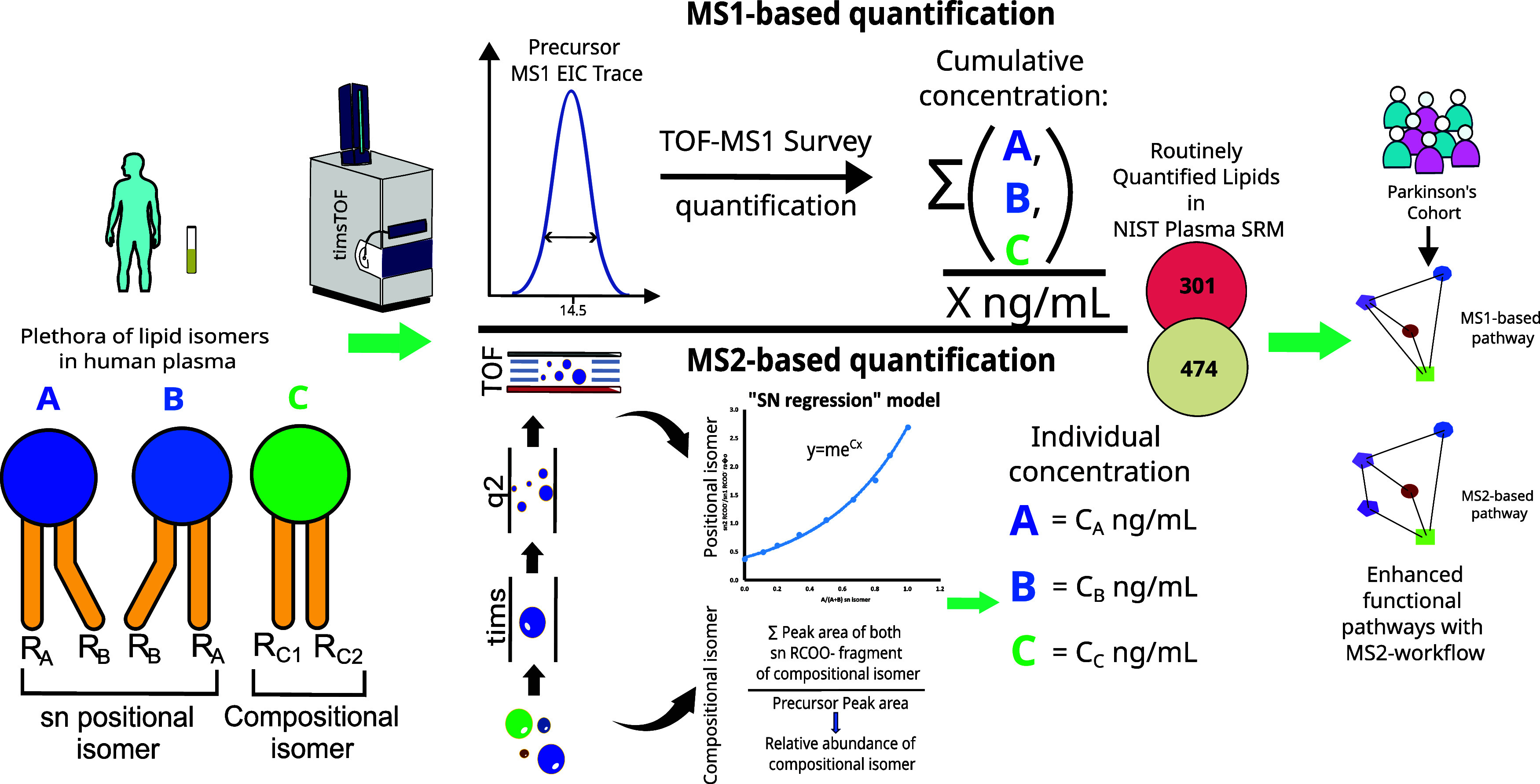
Schematic representation of the experimental workflow used for high-throughput prm-PASEF-based lipidomic profiling in human plasma. The diagram illustrates the differences in identification and quantification of coeluting isomers between MS1- and MS2-based workflows. The prm-PASEF-based approach increases the overall number of routinely profiled lipids in human plasma. The developed framework was employed for lipid biomarker discovery in Parkinson’s disease patients.

### Standard Solutions for Various Isomeric Models

To evaluate the variation of the sn2/sn1 fragment peak area ratio with the acyl chain lengths and the unsaturation degree, a standard mix solution of PA, PC, PE, PG, PI, and PS lipids was prepared at 0.5 μg/mL in methanol. The list of phospholipid standards is shown in Supplementary Data 2.

From here on, the isomer with the acyl chain in the same order as in the molecular species level annotation of the analyte is referred to as the SNA isomer (e.g., PC 14:0/18:0), whereas its other sn positional isomer is referred to as the SNB isomer (e.g., PC 18:0/14:0). For the “SN regression” model, commercially available standards of both the SNA and SNB isomers of PC 32:0, PC 34:0, PC 34:1, PC 36:1, PE 34:1, and PS 34:1 were used. A series of mixtures of all of the aforementioned PC standards containing both SN A and SN B isomers were prepared in the ratio of 1:1, 1:2, 1:4, 1:8, 2:1, 4:1, and 8:1 (SNA:SNB, v/v). Similarly, to estimate the proportion of coeluting isomers with different acyl chains, standards of PC 36:2; PC 18:1/18:1 and PC 18:0/18:2, and PC 36:4; PC16:0/20:4, and PC 18:2/18:2, were used. A stock solution of 1 mg/mL in methanol was prepared for each standard, and subsequently, standard mix solutions were prepared with each of these isomers in different proportions of 1:1, 2:1, 4:1, 8:1, 1, 1:2, 1:4, and 1:8 (v/v).

### Calculations of the sn2/sn1 Ratio

The prm-PASEF resulting peak area of the sn1 and sn2 acyl chain fragments was used to calculate the sn2/sn1 ratio corresponding to individual lipid compositions. For the evaluation of the ratio of sn isomers from coeluting precursors, the sn2/sn1 fragment peak area ratio in the SNA:SNB mix of each of the PC standards was calculated and then averaged across all standards per class to obtain a sn2/sn1 fragment peak area ratio, which is thereby independent of the acyl chain length and unsaturation degree. This procedure was repeated for all of the standard mixtures prepared for this model. For the sake of simplicity, the SNA:SNB ratio was converted to the proportion of the SNA isomer in the whole mixture. The resulting regression curve and coefficient obtained from this curve (Figure 1) were used to calculate the abundance of SNA and SNB in the PLs mixtures as follows:

$$\mathrm{SNA}/(\mathrm{SNA}\;+\;\mathrm{SNB})=\left(\ln\left(\frac{\frac{\mathrm{sn}2\mathrm{COO}\;-\;\mathrm{fragment}\;\mathrm{ion}\;\mathrm{peak}\;\mathrm{area}}{\mathrm{sn}1\mathrm{COO}\;-\;\mathrm{fragment}\;\mathrm{ion}\;\mathrm{peak}\;\mathrm{area}}}{\mathrm{regression}\;\mathrm{coefficient}\;1}\right)\right)/(\mathrm{regression}\;\mathrm{coefficient}\;2)$$

where SNA and SNB are the amounts of SNA and SNB isomers, respectively, and regression coefficients 1 and 2 were obtained by plotting the sn2/sn1 value against the corresponding SNA:SNB ratio

(SNB)=100−(SNA)

Similarly, for estimating the proportion of coeluting FA compositional isomers, the corresponding sn1 or sn2 acyl chains fragment ion peak area of each coeluting isomer is summed up and normalized against the MS1 peak area of the precursor. The compositional lipid isomers are distinguished with the suffix “_Iso” (Supplementary Data 3).

1)
$\mathrm{fraction}\;\mathrm{of}\;A=\left(\left(\frac{\mathrm{sn}1+\mathrm{sn}2\;\mathrm{fragment}\;\mathrm{peak}\;\mathrm{area}\;\mathrm{of}\;\mathrm{isomer}\;A}{P}\right)\right)$

2)
$\mathrm{fraction}\;\mathrm{of}\;B=\left(\left(\frac{\mathrm{sn}1+\mathrm{sn}2\;\mathrm{fragment}\;\mathrm{peak}\;\mathrm{area}\;\mathrm{of}\;\mathrm{isomer}\;B}{P}\right)\right)$

For the TG and DG lipid classes, the characteristic fragment ion [M+NH_4_–(RCOOH+17)]^+^ was used for quantification. This fragment ion can originate from each of the three FAs at different SN positions of the glycerol backbone. Typically, the most intense fragment ion serves as the quantifier ion, unless it is also the most intense fragment ion that is shared with a coeluting TG isomer.

The lipid descriptors and the quantifier ions used for the prm-PASEF analysis of both NIST plasma SRM and serum of the PD cohort are listed in Supplementary Data 3. The negative mode acquisition target list consisted of 224 lipid species with 18 ISTDs; the positive mode contained 143 lipid species with 7 ISTDs. All the targeted lipid species were quantified by normalization to level-2 or level-3 ISTD as defined by LSI guidelines.^34^

prm-PASEF data of the NIST plasma SRM and Parkinson's disease (PD) cohort were processed using Skyline v22.2.^35^ A differential expression analysis (DEA) using limma’s linear modeling function (lmFit)) followed by empirical Bayes moderation (eBayes),^36^ and a t-distributed Stochastic Neighbor Embedding (t-SNE), was performed to visualize the difference between the two groups HC and PD and potential sex specificities with the PD. The significant lipids from DEA were subjected to biosynthetic and functional pathway analysis using BioPAN^37^ and Reactome.^38^

### Method Validation

Limit of Detection (LOD) and Limit of Quantitation (LOQ) were calculated using one lipid standard per class, following the bioanalytical method validation guidelines.^39,40^ For this, a linear concentration range^14^ consisting of 7 serially diluted lipid standard mixtures was used (Supplementary Data 4).

### Phospholipase A_2_ (PLA_2_) Digestion

The phospholipase A_2_ enzyme was used for site-specific digestion of PLs and subsequent performance verification of the SN regression model. The enzyme preferentially cleaves the COO^–^ of the sn2 position of the glycerol backbone, resulting in lyso species containing the sn1 acyl chain of the PL. PLA_2_ digestion was performed for individual PC standards as well as in a mixture. From the ratio of all the resulting lyso species after digestion, the isomeric purity of the original PC standards was calculated.

### Matrix Effect

The matrix effect was evaluated by determining, based on prm-PASEF and sn regression model, the proportion of the commercially available isomer PC 17:0/14:1 d5 relative to its sn congener PC 14:1/17:0 d5 present in a standard mixture as well as in NIST plasma SRM, spiked before and after the extraction (Supplementary Data 6). Additionally, the NIST plasma SRM was spiked with PC 18:1/16:0 (endogenously absent), and its abundance was determined using the SN regression model (Supplementary Data 6).

## Results and Discussion

### prm-PASEF Development for Plasma Lipidome

The prm-PASEF acquisition requires prior determination of essential descriptors of the target molecule, such as RT, *m*/*z*, ion mobility (*K*_0_), and optimal collision energy (eV) for the efficient fragmentation of the targeted molecules. To benchmark and optimize the prm-PASEF method, already-known 4-dimensional (4D) descriptors of plasma lipids determined in prior work^14^ were used to generate a preliminary target list (Supplementary Data 5). The isolation width of 1 Da was found optimal to avoid interference from isotopologues of neighboring peaks and streamline downstream data processing. Given the good chromatographic reproducibility, an RT range of 60 s sufficed for prm-PASEF acquisition with no missed targets in the replicate analysis. A collision energy of 45 eV was selected as the optimal value for prm-PASEF fragmentation in both positive and negative modes. For PA, PI, and PS, the precursor ion remained the main ion in the MS2 spectrum, followed by the FA carboxylate ions. The latter ion type is the main fragment ion of the other PL classes. Hence, for PA, PI, and PS, a collision energy value of even higher than 45 eV can be used.

### TIMS Survey vs prm-PASEF

Targeted analysis on HRMS, such as prm-PASEF, is expected to have superior sensitivity and data quality to untargeted acquisition with DDA due to more scan cycles per target. This results in an increased number of data points and enhanced selectivity using narrow isolation ranges within 2-dimensional space of mobility and RT.^32^ The MS2 spectrum for PC 36:2 in NIST plasma SRM was specifically used for this evaluation. The DDA-PASEF data resulted in only one PASEF scan for this precursor, with *m*/*z* 279.2350, 281.2485, and 283.2641 Da corresponding to carboxylate ions of C18:2, C18:1, and C18:0, respectively, indicating the presence of both isomers PC 18:0_18:2 and PC 18:1_18:1. Acquisition via the TIMS-DDA-PASEF mode was unable to resolve the two isobars into distinct features for subsequent individual species quantification. Only the presence of these isomers in the sample is determined from the overlapped DDA-PASEF MS2 spectra, but they cannot be distinctly quantified. Retrieval of isomer-specific fragment ions and their intensities from the DDA-PASEF scan data is not readily feasible by software tools and also not reproducible enough when done manually from raw data to ensure reproducible quantification output of each isomer. prm-PASEF acquisition of the PC 36:2 precursor and subsequent software-based extraction of precursor-fragment ion peak area rendered distinct MS2 spectra: (i) one PASEF scan containing only FA carboxylate fragment ions at *m*/*z* 279.2350 and 283.2641 Da and (ii) another PASEF scan with only fragment ions at *m*/*z* 281.2485 Da. Also, the abundance of prm-PASEF precursor peak was higher compared to the ones obtained by MS1 survey TIMS-DDA-PASEF. The ratio of the sn2 RCOO^–^ and sn1 RCOO^–^ ions was found to be similar between the two acquisition modes for most of the PLs across different lipid classes (Figure 1, Supplementary Data 2).

The developed prm-PASEF method was validated using NIST plasma SRM (*n* = 3). Initially, 209 lipids with 23 ISTDs covering major lipid classes such as ceramide (Cer), fatty acid ester of free hydroxyl fatty acid (FAHFA), hexosyl ceramide (HexCer), lysoglycerophosphocholine (LPC), lysoglycerophosphoethanolamine (LPE), PC, plasmanyl PC (PC O-), PE, PI, PS, and sphingomyelin (SM) were included in the prm-PASEF acquisition list. The average coefficient of variation (CV) for quantified values was less than 30% for each of the 11 lipid classes except the PS lipid class.

## Method Validation

Both LOD and LOQ values for each lipid class, except for PI and PS lipid classes, were found to be superior to those obtained with the MS1-based workflow (Supplementary Table 1, Supplementary Data 4). For all the lipid classes, the calculated LOD and LOQ were lower than the lowest analyzed concentration points in the serial concentration range. Only for PS and Cer, the LOQ was found to be higher than the lower concentration point (Supplementary Table 2, Supplementary Data 4). For each lipid class, the linearity was evaluated with an *r* ^2^ coefficient of >0.99 (Supplementary Data 4).^54^

### ISTD Performance in prm-PASEF Quantification

For each of the lipid classes, odd acyl chain lengths and deuterated ISTD were evaluated for the prm-PASEF performance. The use of deuterated ISTDs resulted in a CV better than that of the odd acyl chain length ISTD for almost every lipid class. The ISTD resulting in the lowest CV value of the normalized peak area of the targeted analyte was chosen for all subsequent applications (Supplementary Table 1).

### Variation of the sn2/sn1 Ratio with Varying Lipid Structures

For the majority of lipid classes, the sn2 RCOO^–^ (sn2) fragment is usually more intense than the sn1 RCOO^–^ (sn1) fragment. However, factors such as the degree of unsaturation and FA chain length can cause variations in the peak area ratio of the sn2/sn1 fragments of PLs^18^ (hereon referred to as the sn ratio). To evaluate these variations, the sn ratio was calculated for pure standards of PC, PS, PG, PE, and PI (Supplementary Data 2).

The ratio was found to be higher (∼3) for lipids with C14 acyl chains in the sn1 position compared with C16 or C18. For the C16 or C18 acyl chains at the sn1 position, the ratio remained relatively consistent between 2.1 and 2.6, especially for PCs (Supplementary Data 2).

A similar trend was observed for the acyl chains at the sn2 position, where the ratio was found to be negatively correlated to the increase in the acyl chain length. Also, this correlation was found to be strongly dependent on the number of C atoms at the sn1 position. For the same number of C atoms and unsaturation at the sn2 position, the sn ratio decreased with an increase in the number of carbon atoms at the sn1 position.

Due to the unavailability of standards, we could only evaluate the effect of unsaturation at the sn2 position. The sn ratio was found to be positively but poorly correlated (*r* ^2^ = 0.35) with the amount of unsaturation at the sn2 position. These trends were observed for PS, PE, and PG as well, irrespective of the abundance of the sn2 RCOO^–^ fragment ion compared to its sn1 counterpart (Supplementary Data 2). These observations were also confirmed by a comparison of the sn ratio of the MS2 spectra obtained through DDA-PASEF acquisition (Supplementary Data 2).

### Development of the SN Regression Model

A set of 4 PC molecular compositions varying in their acyl chain and total unsaturation, namely, PC 14:0_18:0, PC 16:0_18:0, PC 16:0_18:1, and PC 18:0_18:1, was mixed in eight different proportions with their corresponding sn positional isomer. The resulting sn ratio from each of the mixtures suggests that at least for the PC class the sn ratio of this mixture varies very little with the acyl chain length and/or unsaturation. Therefore, for each PC molecular composition with differing concentrations of the respective SNA or SNB isomer, an average of the sn ratio was calculated. This average value was used as the sn ratio value for that particular SNA isomer concentration. A plot of the sn ratio for all 8 SNA isomer concentration points resulted in a regression curve with an excellent exponential relation (Figure 1, Supplementary Data 6). These regression coefficients can be used to calculate the SN isomeric proportion of virtually all PC lipids. This workflow is termed the “SN prediction model”.

The same procedure was applied for the PE and PS classes. However, for these classes, the sn ratio was derived from only one molecular composition (16:0_18:1). The regression coefficients 1 and 2 were 0.40 and 1.91 for PC, 0.38 and 1.66 for PE, and 0.72 and 1.33 for PS, respectively. The SN prediction model was first used to determine the purity of some of the commercially available PC standards, such as PC 16:0/18:1, PC 16:0/18:2, PC 16:0/20:4, PC 18:0/18:1, and PC 18:0/18:2, and benchmark our model against purity specifications of the standards. The calculated isomeric purity (in %) for each of the standards was found to be between 93 and 99% (Supplementary Data 6). To highlight the performance of the model, each of the standards was injected at different concentrations ranging from 2.4 to 24 pmol. Each concentration point resulted in a similar isomeric purity for each investigated standard. The isomeric purity of PC 16:0/18:1 and PC 18:0/18:1 predicted by the SN model, however, deviated slightly, by 5 and 0.53%, respectively, from the previously reported values of 88 and 96%.^16^

To further validate the SN prediction model, the purity of the PC standards was determined by the phospholipase A_2_ (PLA_2_) enzymatic digestion of the PC standards^23^ individually and in a mixture. The PLA_2_ digestion resulted in a (%) isomeric purity of the standard in good accordance with the values predicted by the SN prediction model for all the PC standards (CV < 2%), as shown in Supplementary Table 3 and Supplementary Data 7. PLA_2_ digestion was found to have >99% digestion efficiency as no precursor trace was observed in the digested mixtures (Supplementary Figure 1). This was observed regardless of the concentration of the mixture used, 1.5 pmol to 6 pmol. To further validate this model, a blindfold study was carried out in which two mixtures (MIX A and MIX B) with unknown amounts of both the sn positional isomers of PC 32:0, PC 34:1, PC 36:1, PE 34:1, and PS 34:1 were prepared. As shown in Supplementary Table 3, the model was able to predict the proportion of each isomer with an accuracy of 97%.

The isomeric content of the externally spiked commercially available ISTD PC 17:0/14:1 d5 was determined using the SN regression model to be approximately 85% in both a standard mixture and NIST plasma SRM spiked before and after extraction, suggesting an insignificant matrix effect on the model. This was further verified by determining the spiked abundance (20%) of endogenously absent PC 18:1/16:0 in the NIST plasma SRM (Supplementary Data 6).

### Lipid Compositional Isomer Quantification

Other than sn positional isomers, there also occur instances where coeluting isobaric compositional isomers with differing acyl chains cannot be distinctly quantified. Even though each coeluting precursor will produce diagnostic fragments in MS2 for identification, only MS2-based quantification can be applied to distinctly quantify such species of interest in biological matrices.^22,41–43^

The MS2 quantification for coeluting Pls isomers is, again, based on the observed sn RCOO^–^ fragment ions in the negative mode. We used coeluting isomers of PC 36:2, namely, PC 18:0/18:2 and PC 18:1/18:1, and PC 36:4, namely, PC 16:0/20:4 and PC 18:2/18:2, to evaluate the model. The peak area corresponding to the sn1 and sn2 diagnostic fragments of each of these coeluting isomers was retrieved and then used to estimate the proportion of each of the coeluting compositional isomers. The precursor ratios from seven mixtures with different concentrations of each isomer of the PC 36:2 and PC 36:4 species were predicted with an accuracy of 90–110%, with a few exceptions (Supplementary Data 8). Since no additional changes to the prm-PASEF analytical settings are needed for this MS2-based quantification, our workflow can be reliably used to quantify the coeluting isomers with differing acyl chain lengths in addition to sn positional isomers, thus expanding the quantitative coverage capabilities of complex lipidomes.

The quantification of sn coeluting TGs isomers is especially challenging.^44^ The same FA can be shared between two or all of the sn1, sn2, or sn3 positions. Moreover, the ratio between the [M-sn RCOO^–^] fragment ions from sn1, sn2, and sn3 FAs in TGs varies substantially, e.g.: TG 15:0_16:0_16:0, TG 15:0_14:0_18:0, and TG 15:0_15:0_17:0 in which case, the use of [M-16:0 COO^–^] or [M-15:0 COO^–^] renders overexpressed concentrations of such TGs. Also, the 20:4 FA fragment ion was found to be more intense than other FAs, regardless of the sn position. Therefore, establishing a reliable sn isomer fragmentation model for TGs requires prospective deconvolution and more standards. Consequently, here, only the coeluting isomers with a dissimilar FA chain to the rest of the coeluting isomers were quantified. For this, the [M-sn RCOO^–^] fragment ions with the highest peak area was used for quantification.

### Deeper Profiling of NIST Plasma SRM

We used NIST plasma SRM to demonstrate the method’s performance in human plasma and benchmark the method against the TIMS survey quantitative coverage.^45–47^ In our previous studies,^14^ we reported the routine quantification of 359 lipids using the TIMS survey, whereby only noncoeluting isomers were distinctly quantified. Here, using the prm-PASEF combined with the “SN prediction” model, a deeper routine quantitative profiling of the NIST plasma SRM was obtained, up to 481, which included 68 PC, 15 PE, and 93 TG coeluting isomers, respectively. Nearly 70% of the MS2-based quantified lipids showed <42% CV over 32 NIST plasma SRM replicates. For lipid species with CV >40%, an average of 12 replicates out of 32 were found to be outliers. The outliers were identified based on the interquartile range (IQR) with a stringent threshold of 0.1 unit. Most of the outliers were low-abundant molecules for which fragment ions’ intensities are expected to vary more than for the high-abundant molecules, resulting in lower reproducibility of MS2-based quantification. Also, lower reproducibility can arise for isobaric lipids sharing a common FA chain. For instance, PC 17:0/20:4 and PC O-18:0/20:4 share FA chain, C20:4, which will be the chosen quantifier ion transition for both the isobars, also due to its higher abundance. Also, they share a partial overlap in their scheduled RT window (also for PE 16:0/22:6 and PC 17:0/14:1). Such factors might lead to inaccurate extraction of the precursor-to-quantifier ion transition for the isobaric precursors. The average quantified values (*n* = 32) (excluding outliers) for each of the features are shown in Supplementary Data 3.

In NIST plasma SRM, 89% of the identified PCs were found to contain an SNB positional isomer. As discussed above, the model predicted the sn positional isomeric ratio with an accuracy of ± 20%; so, the lipid species for which the model predicted SNA isomer to be >100% were considered to be isomerically pure. Similarly, the “SN regression” model for PE resulted in the quantification of 12 positional isomers.

Comparison between MS1-suvery and MS2-based quantification resulted in a ratio value of MS2 normalized value/MS1 normalized between 0.7 and 1.3. However, certain lipids exhibit greater differences between the two quantified values (Figure 2). For instance, PC 17:0/18:2 and the coeluting isomer PC 17:1/18:1 contain each a substantial percentage of the other sn positional isomers (i.e., PC 18:2/17:0 (17%) and PC 18:1/17:1 (45%)). These differences might arise from several factors, including the fragmentation efficiency of the precursor or the presence of a coeluting isomer, etc. Since for the MS2-based quantification of each isomer is accounted for individually, the resulting MS2-based quantified values are expected to differ from the MS1 precursor ion-based quantified values (Figure 2).

**Figure 2:**
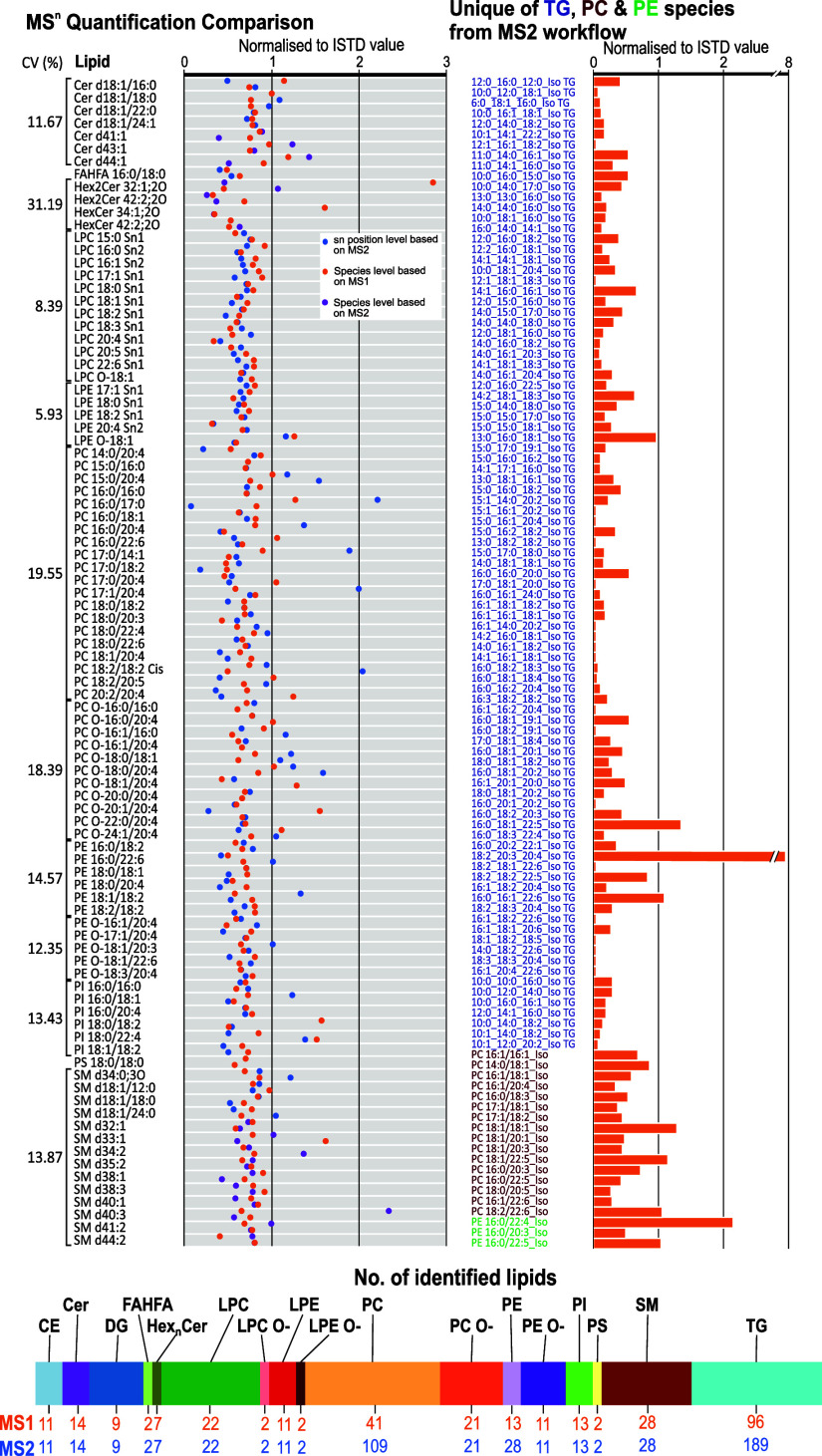
Comparison of the identified and quantified lipid levels in NIST plasma SRM by normalization to level-2 or level-3 ISTDs at the species level—based on MS1 (orange), species level (violet), and sn position level (blue) based on MS2 workflow. It also illustrates the normalized values to ISTD of unique lipid species for PC (reddish purple), PE (bright green), and TG (violet) lipid classes via prm-PASEF.

Overall, 14 Cer, 2 FAHFA, 7 Hex_*n*_Cer, 22 LPC, 2 LPC O–, 11 LPE, 2 LPE O–, 109 PC, 21 PC O–, 28 PE, 11 PE O–, 13 PI, 2 PS, and 28 SM identified in negative polarity and 189 TGs, 11 CEs, and 9 DGs lipids were quantified exclusively using the prm-PASEF and isomeric model in NIST plasma SRM (Figure 2).

### Method Applicability: The Case of Parkison’s Disease

All the 481 lipid targets quantified using both the MS1-based precursor ion peak area and the MS2-based quantifier ion peak area were combined to evaluate the serological lipidomic phenotype of the controls (HC) and patients with Parkinson’s disease (PD) (Supplementary Data 9). An overall upregulation of LPC species due to enhanced PLA_2_ activity was evidenced in Parkinson’s disease patients (PD) (Figure 3) with both MS1- and MS2-based quantification strategies. The LPC upregulation is significantly higher in male PD patients compared with female PD patients (Figure 3). The DEA revealed 148 significant lipids out of 315 lipids for MS1-based values and 90 significant lipids out of 408 lipids for MS2-based values (Figure 4, Supplementary Data 10). Forty three lipids, mostly from LPC and TG lipid classes, were found to significantly differ between male and female groups.

**Figure 3:**
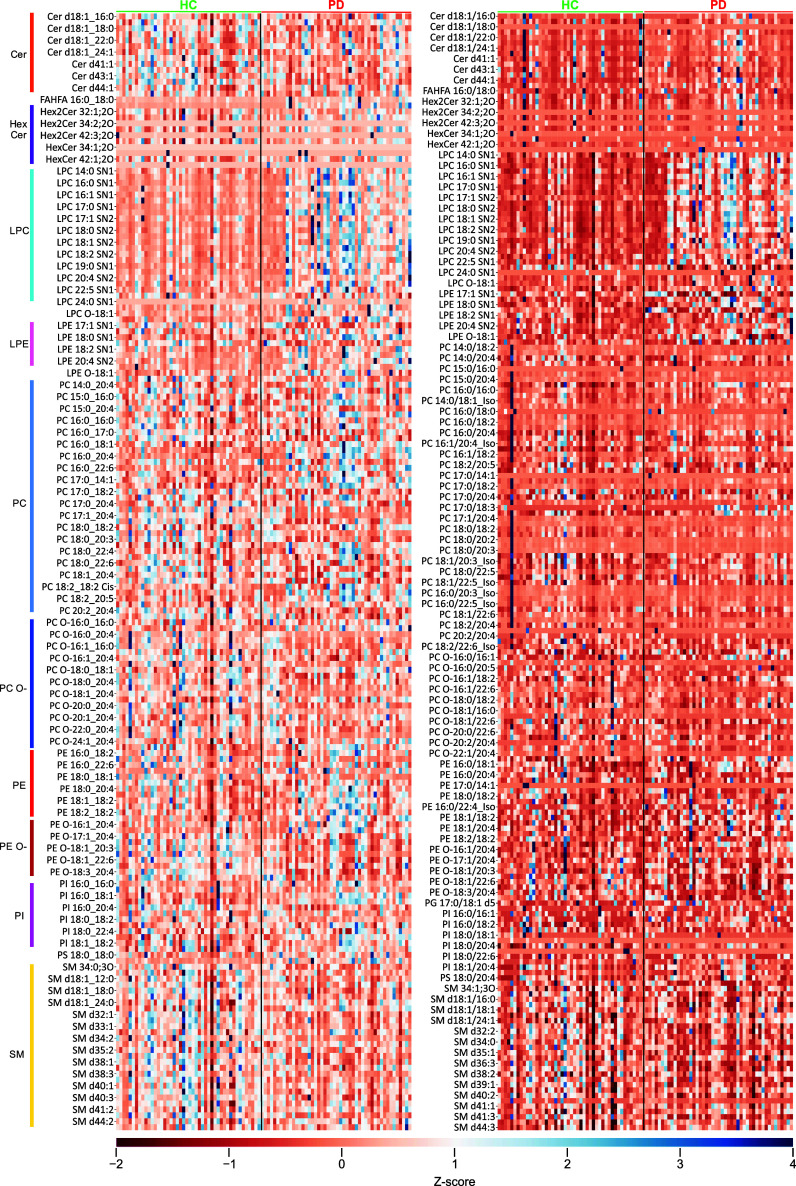
z-Score heatmap showing the enhanced PLA_2_ activity in Parkinson’s disease patients (PD (in red bracket) compared to the controls (HC (in green bracket) leading to overall upregulation of lysoglycerophosphocholines (LPC) in PD with both the MS1 (left) and MS2-based (right) workflow. The z-score scale extends from red (−2) to blue (4).

**Figure 4:**
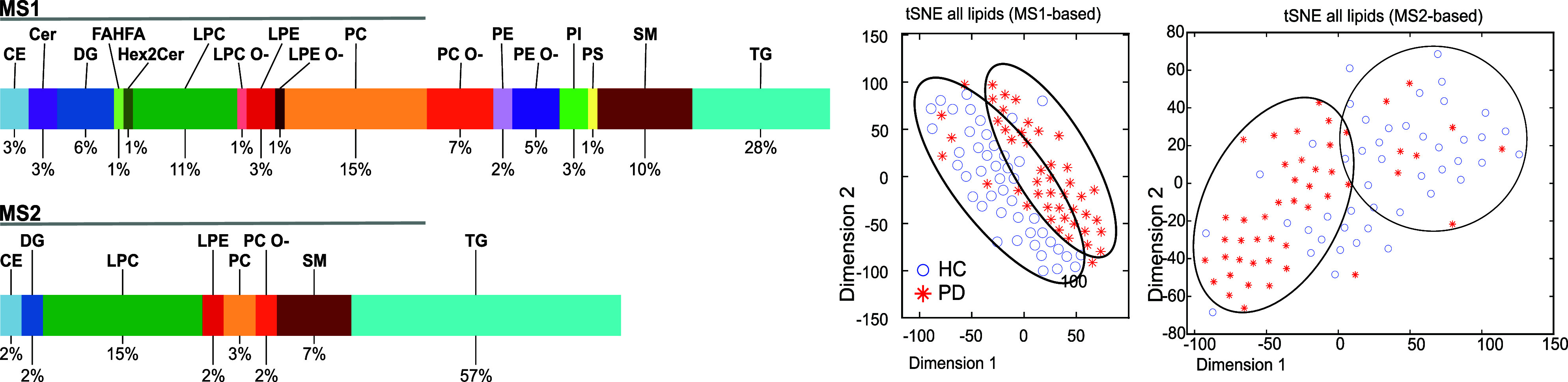
Stacked bar chart showcasing percentage (%) of significant lipid species in both MS1- and MS2-based workflows by differential expression analysis. The t-SNE plot with all the quantified species in the Parkinson’s cohort, with clear distinction of both groups in the MS1-based workflow and with partial overlap in the MS2-based workflow.

Fifty lipid species were differentially expressed between HC and PD patients, regardless of the quantification strategies. The higher number of significant features from MS1-based quantification compared to the MS2-based quantification is attributed to the lower yield of fragment ions of the low-abundance molecules (Supplementary Figure 2). Of note, for some of the coeluting clusters in the TG class, the lipid species found to be significant were solely identified from MS2-based strategies (Supplementary Table 5). This highlights the role of prm-PASEF for deeper quantitative profiling in a biological matrix. The t-SNE analysis (Figure 4) also showed a separation between the HC and PD using both the MS1- and MS2-quantified values. The HC and PD group separation is better using the MS2 than MS1-based data (Figure 4), attesting to the discriminative capability of precise structural annotation and quantification of lipids and ultimately suggestive of the important functional role of these lipids.

The biosynthetic pathways resulting from using BioPAN software from the MS1-based quantification reflect biological information based only on the molecular species level in lipids, hence including clusters of isomers (Figure 5), whereas MS2-based quantification renders information on the lipid species at the sn position level and FA content level.

**Figure 5:**
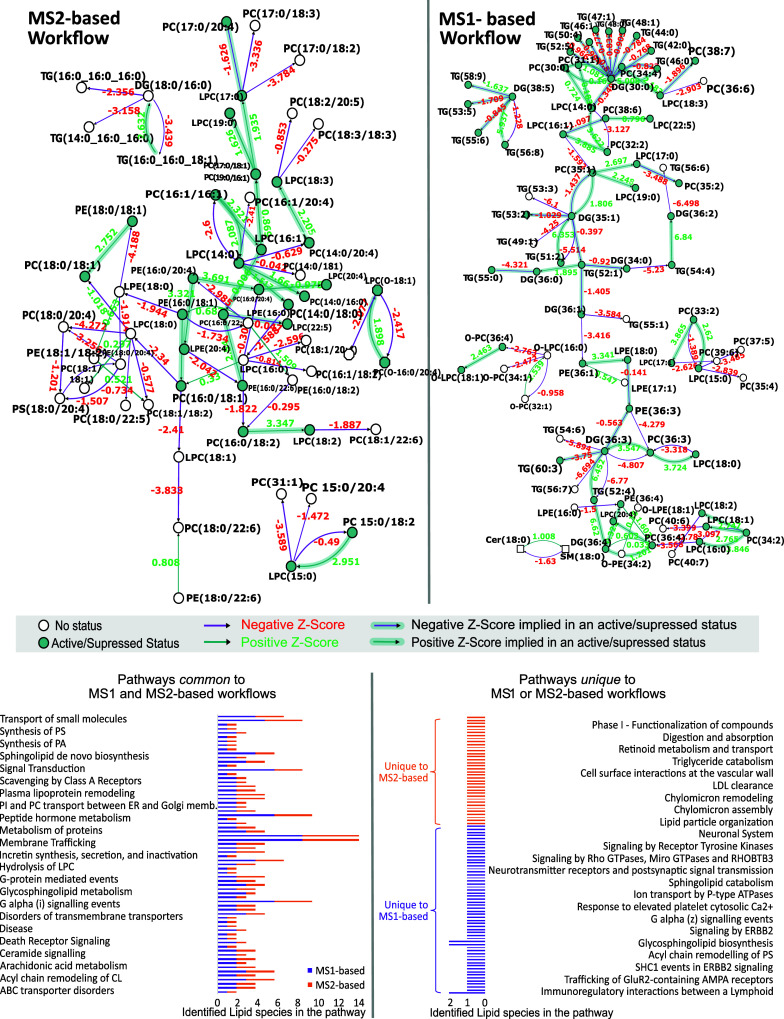
Biosynthetic pathway results using BIOPAN from significant lipid features in HC and PD samples from both MS1- and MS2-based workflows. Each pathway reaction is colored as either purple or green, whereas the z-score in red and green are based on its negative and positive correlation, respectively, between the two groups, HC and PD. The active/suppressed status of a lipid species is indicated by a green-filled circle. An empty-filled circle indicates no status for that lipid species in the pathway. Bottom half of the figure shows the common (left) and unique (right) pathways between the two workflows resulting from Reactome’s online tool.

This, in turn, allows us to understand and follow these pathways at a specific FA level. For instance, the biosynthetic conversion of LPC 14:0 to PC 32:2 is deduced from MS1 data. The fatty acyl content and sn-configuration of PC 32:2 can only be determined by MS2. Subsequently, coeluting fatty acyl-derived isomers of the PC 32:2 can only be individually quantified using MS2 fragments, such as the case here with prm-PASEF and compositional and/or configurational isomeric models. Therefore, the conversion of PC 32:2 to LPC 14:0 can be unambiguously evidenced only if MS2 data demonstrate the presence of saturated myristic acid FA containing an isomer in the cluster of PC 32:2. Accordingly, just based on MS1 data, such a lipid conversion as assigned by lipid conversion software tools would not be unambiguous and/or ultimately accurate. Similarly, the LPC 16:1 to PC 32:2 conversion can only be accurately identified if the coeluting PC 16:1/16:1 isomer in the cluster of PC 32:2 is quantifiable by an MS2-based workflow. Due to this specificity in sn position annotations, the enzymatic role of PLA_2_ in PD can be more precisely elucidated and coupled to FA metabolism rather than using only lipid class or sum composition. Accurate estimation of the LPC/PC ratio for individual FA compositions allows a more accurate insight into FA-specific pathways and remodelling in a disease condition. This enables inference of potential preferential enzyme substrate specificity under a specific disease condition. The LPC/PC ratio can help in distinguishing Parkinson’s patients from controls and, importantly, can be used to better tailor the diagnostic concentration ranges of lipids to female and male patients (Supplementary Figure 3).

The z-score value, obtained for each reaction between the two groups, explained the differences in the lipid metabolism between HC and PD (Figure 5). Ascribing specific lipid reactions to the FA content as afforded by the MS2-based strategy here, rather than using (sub)­class conversion without regard to the specific FA content as typically seen with MS1-based quantification, brings new information on disease-affected pathways.

The functional pathway using Reactome indicated several potential pathways relevant to Parkinson’s condition: plasma lipoprotein assembly, opioid signaling, G-protein mediated events, etc., exclusively identified via prm-PASEF workflow (Figure 5), while 68 pathways are commonly unravelled by both methods.

These pathways can be used to better prioritize enzymatic targets and substrates for subsequent validation and therapeutic design, rather than relying strictly on lipid class-based pathway analysis, which results in collapsing isomeric species into lipid sum composition when MS1 survey quantification is used.

## Conclusions

The integration of prm-PASEF with an SN regression model enhances the quantification of FA compositional and sn position isomers, broadening lipidome coverage in clinical profiling. This is crucial for distinguishing the functional roles of lipid isomers, which are often masked in current software that assigns functions to broader lipid classes. The approach is applicable across various biological sample types and conditions involving lipid metabolism.

In Parkinson’s disease, we observed dysregulation of PCs, LPCs, and TGs, aligned with known enzymatic and inflammatory changes. TG compositional isomers contributed substantially to differentiation of PD from HC, emphasizing FA metabolism’s role. Lipid alterations in PD were also tied to immune signaling (TLR, calcium), neurodegeneration,^48,49^ and ER-Golgi function.^50–52^ Importantly, lipidomic profiling revealed sex- and age-specific patterns in PD distinct from normal aging, suggesting disease-specific pathways.^53^

In summary, the prm-PASEF workflow with isomer modeling offers deeper insights into lipid function and pathology, advancing biomarker discovery and disease mechanism studies.

## Supplementary Material





























## Data Availability

All the associated experimental data will be provided on request. “This material is available free of charge via the Internet at “http://pubs.acs.org” as indicated in the main text.

## Notes

Base peak chromatograph of PC standards before and after PLA_2_ digestion, differences between the MS1- and MS2-quantified values of significant lipids with PD, LPC/ΣPC ratio using MS1 or MS2 for various lysoglycerophosphocholines with PD, z-score heatmap of TG with PD, list of internal standards, limit of detection and limit of quantification, phosphatidylcholine standards purity, demographic data, significant features with PD based on t-SNE analysis, materials and methods, chemicals and solvents, lipid standards, sample preparation, TIMS PASEF acquisition, method validation, data processing, standard solutions for various models, calculations of the sn2/sn1 ratio, PLA_2_ digestion, calculations of the standard purity after PLA_2_ digestion, method applicability in PD, software, and statistics and visualization (PDF)
Content of supplementary data (PDF)
Acquisition and processing transition list with all the lipid species targeted in negative and positive ion modes for the prm-PASEF method (XLSX)
sn2/sn1 ratio from lipid species of major lipid classes obtained with both prm-PASEF and DDA-PASEF acquisition strategies (XLSX)
Positive and negative descriptors for every lipid species such as RT, precursor *m*/*z*, product *m*/*z*, retention time window, and ion mobility (V.s/cm^2^) used for data processing of prm-PASEF data; quantified values of all lipid species in respective ionization modes, as mentioned in the article, in both MS1- and MS2-based workflows with the relative proportion of “sn positional” isomer and coeluting compositional isomers exclusively indicated by suffix “_iso” (XLSX)
Calculations of LOD, LOQ (in picomolar (pM) concentration), and accuracy (in %) as shown in the article with a representative lipid standard from every lipid class (XLSX)
Precursor target list with target lipid species and the corresponding descriptors used for the prm-PASEF method acquisition of the glycerophospholipid mixture, also used as a QC for every experiment; the glycerophospholipid mixture consisted of a few lipid species from every lipid class (XLSX)
“SN prediction model” for PC, PE, and PS lipid classes; calculations of the isomeric abundance of the “sn positional” isomer for various lipid species from PC, PE, and PS lipid classes in a standard mixture and NIST human plasma SRM; showcasing of the matrix effect with calculations of abundance of isomerically pure commercially available internal standard PC 17:0/14:1 d5 in the standard mixture, spiked in NIST plasma before and after extraction (XLSX)
Calculations of the isomeric purity of PC standards in various concentration mixtures based on the PLA_2_ assay; abundance of the sn positional isomers (SNA and SNB) for commonly occurring glycerophosphocholine (PC) standards calculated using phospholipase A2 (PLA_2_) enzymatic activity, “SN regression” model, and that reported in the literature (XLSX)
Calculations of the isomeric abundance of PC compositional isomers in various concentration mixtures (XLSX)
Quantification values of lipid species for all samples in Parkinson’s cohort; Parkinson’s cohort consists of two groups HC (healthy control) and PD (Parkinson’s Disease) with 33 female and 59 male participants (XLSX)
List of significant features arising from t-SNE analysis between healthy control (HC) and Parkinson’s disease (PD) patients for both MS1- and MS2-based quantified values, the data shows the significant features for both unimputed (original) and imputed data with missing values for certain lipid species, and the data also show the significant features when both age and sex are considered as variable for t-SNE analysis (XLSX)
Reporting checklist for NIST Plasma, ILS (PDF)
Reporting checklist for Parkinson, ILS (PDF)
