# Supplementary material for: prm-PASEF-Based Quantification and Isomeric Model for Extended Coverage of Human Plasma Lipidome in Parkinson’s Disease
Source: Anal Chem. 2025 Oct 27;97(44):24295–305. doi: 10.1021/acs.analchem.5c02340 (PMC12613151; doi:10.1021/acs.analchem.5c02340)
Supplement: Supplementary file 1 [file ac5c02340_si_001.pdf]

prm-PASEF-based quantification and isomeric model for extended coverage of human plasma lipidome in Parkinson's disease

Dhanwin Baker[a], Gabriel Gonzalez Escamilla[b], Daniel Janitschke[c], Yvan Devaux[d], Nils Schröter[e], Sergiu Groppa[f], Laura Bindila[a*]

Author

[a]Clinical Lipidomics Unit, Institute of Physiological Chemistry, University Medical Center of the Johannes Gutenberg University Mainz, Duesbergweg 6, 55128, Mainz, Germany

E-mail: dhabaker@uni-mainz.de; ORCID ID: 0000-0002-4470-3379

[b]Gabriel Gonzalez Escamilla (GGE) - Department of Neurology, Universitätsklinikum des Saarlandes, Kirrberger Straße 100, 66421, Homburg, Germany

E-mail: Gabriel.Gonzalez@uks.eu; ORCID ID: 0000-0002-7209-1736

[c]Daniel Janitschke (DJ) - Department of Neurology, Universitätsklinikum des Saarlandes, Kirrberger Straße 100, 66421, Homburg, Germany

E-mail: daniel.janitschke@uks.eu; ORCID ID: 0000-0001-8966-0184

[d]Yvan Devaux (YD) - Cardiovascular Research Unit, Department of Precision Health, Luxembourg Institute of Health, 1 A-B Rue Thomas Edison, 1445 Strassen, Luxembourg

E-mail: yvan.devaux@lih.lu; ORCID ID: 0000-0002-5321-8543

[e]Nils Schröter (NS) – Clinic for Neurology and Neurophysiology, Universität Klinikum Freiburg, Breisacher Straße 64, 79106, Freiburg Germany

Email: nils.schroter@uniklinik-freiburg.de; ORCID ID: 0000-0002-3833-9822

[f]Sergiu Groppa (SG) – Department of Neurology, Universitätsklinikum des Saarlandes, Kirrberger Straße 100, 66421, Homburg, Germany

E-mail: sergiu.groppa@uks.eu; ORCID ID: 0000-0002-2551-5655

[a*]Laura Bindila (LB) - Clinical Lipidomics Unit, Institute of Physiological Chemistry, University Medical Center of the Johannes Gutenberg University Mainz, Duesbergweg 6, 55128, Mainz, Germany
*E-mail: bindila@uni-mainz.de Tel: +4961313925794; ORCID ID: 0000-0001-5538-2008

KEYWORDS. Parallel Reaction Monitoring (prm), Isomeric Lipids, Trapped Ion mobility spectrometry (TIMS), Parkinson's Disease, Lipidomics

For Table of Contents Only

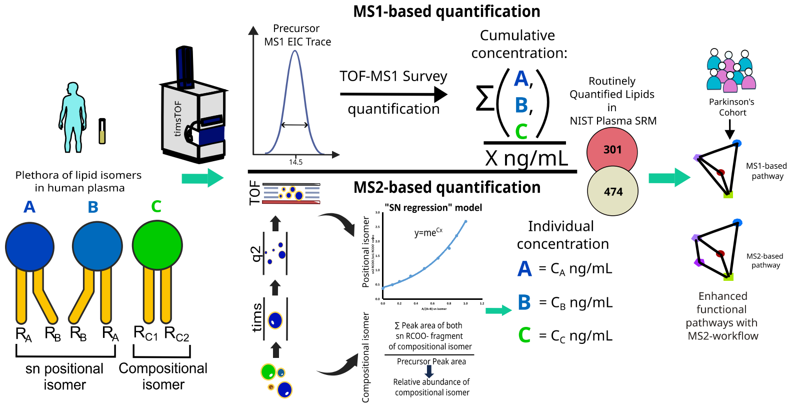

Supplementary Figure 1: Base peak chromatograph (BPC) for a mixture of PC standards before (orange) and after (Blue) phospholipase A_2 (PLA_2) enzymatic effect. After PLA_2 digestion, the mixture has an increased amount of lysoglycerophosohocholines (LPC) and no traces of undigested glycerophosphocholine (PC) species.

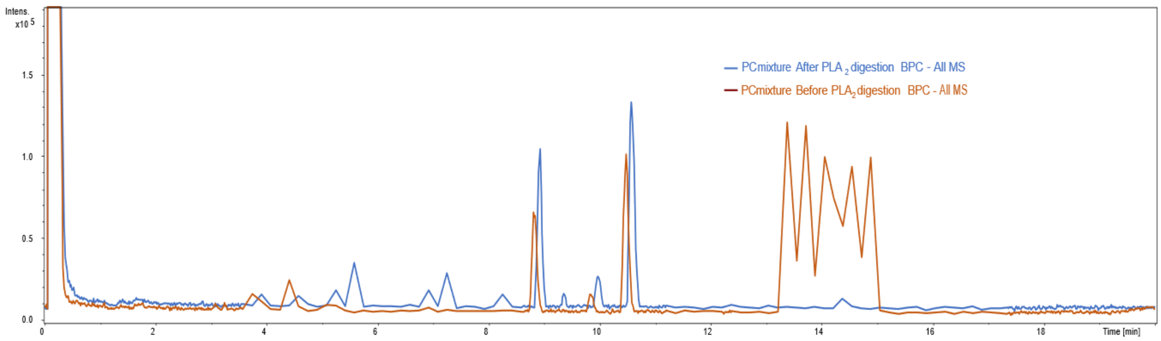

Supplementary Figure 2: Differences between the MS1 (blue) and MS2 (orange) quantified values (log_{10}) for lipid molecules found significant only with the MS1-based quantified values in both healthy controls (HC) and Parkinson's patient (PD). The low abundance of these significant features in the plasma samples is especially highlighted during the MS2-based quantification and therefore not resulting as significant features differentiating the two groups HC and PD for MS2-based quantification.

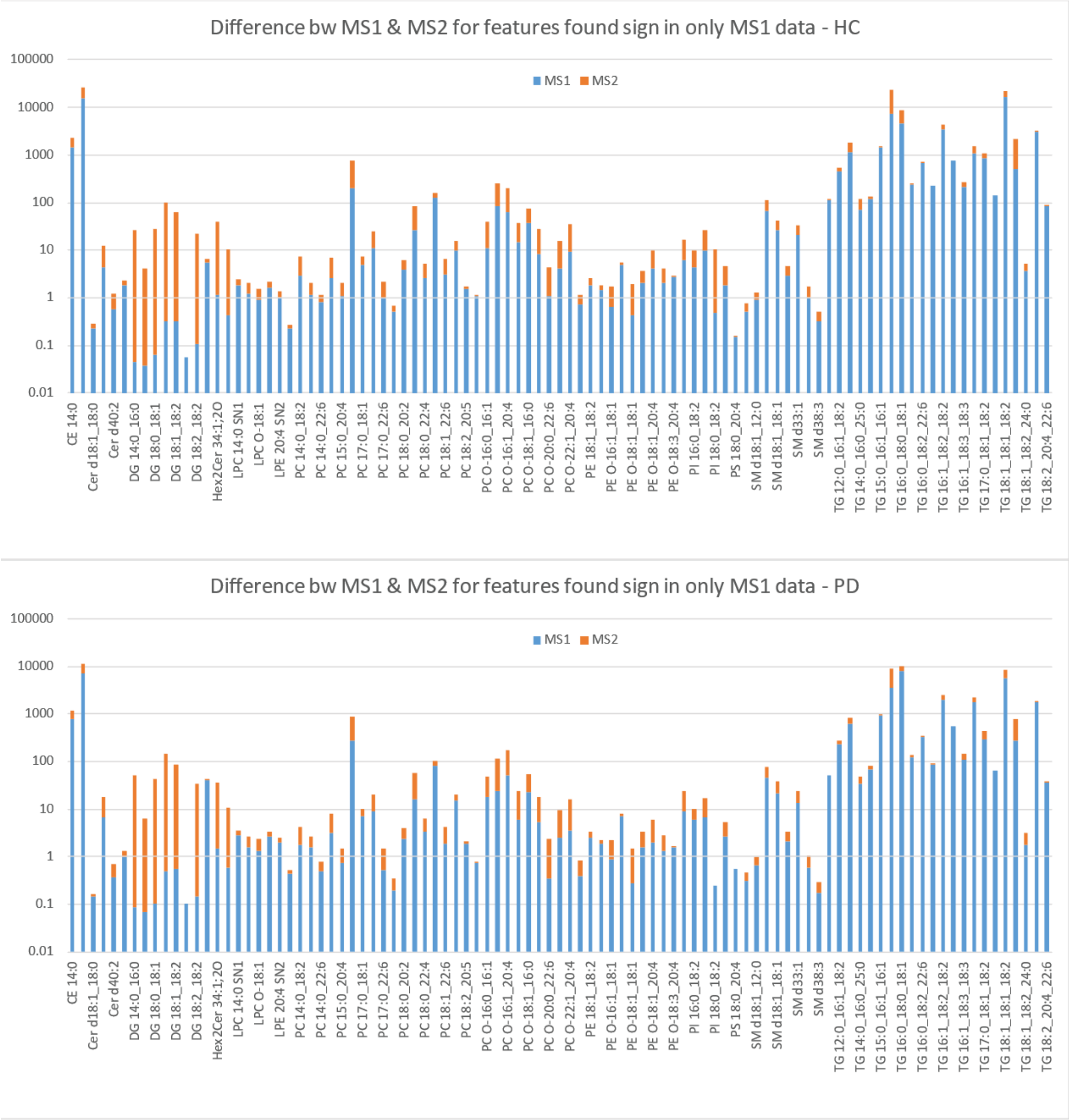

Supplementary Figure 3: The LPC/ΣPC ratio for various lysoglycerophosphocholines (LPC) quantified with MS1 (Blue) and MS2 (Orange) based quantified values in PD samples. The extensive coverage of phosphocholines with all the possible co-eluting compositional isomers with MS2-based quantification results in accurate and higher LPC/ΣPC ratio for MS2-based quantified values as compared to MS1-based quantified values.

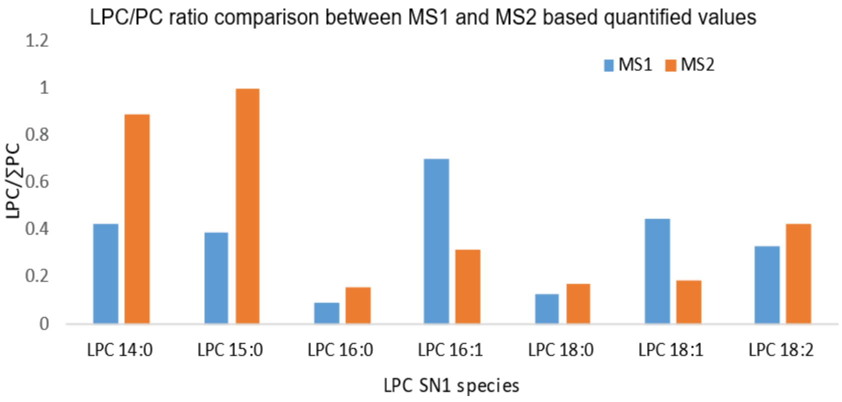
LPC/PC ratio comparison between MS1 and MS2 based quantified values
MS1
MS2
LPC/∑PC
1.2
1
0.8
0.6
0.4
0.2
0
LPC 14:0
LPC 15:0
LPC 16:0
LPC 16:1
LPC 18:0
LPC 18:1
LPC 18:2
LPC SN1 species

Supplementary Figure 4: z-score heatmap showing downregulation of certain TG species in PD (red) compared to HC (Green) at molecular species level with both the MS1 (left) and MS2-based (right) workflow. The z-score scale extends from red (-2) to blue (4).

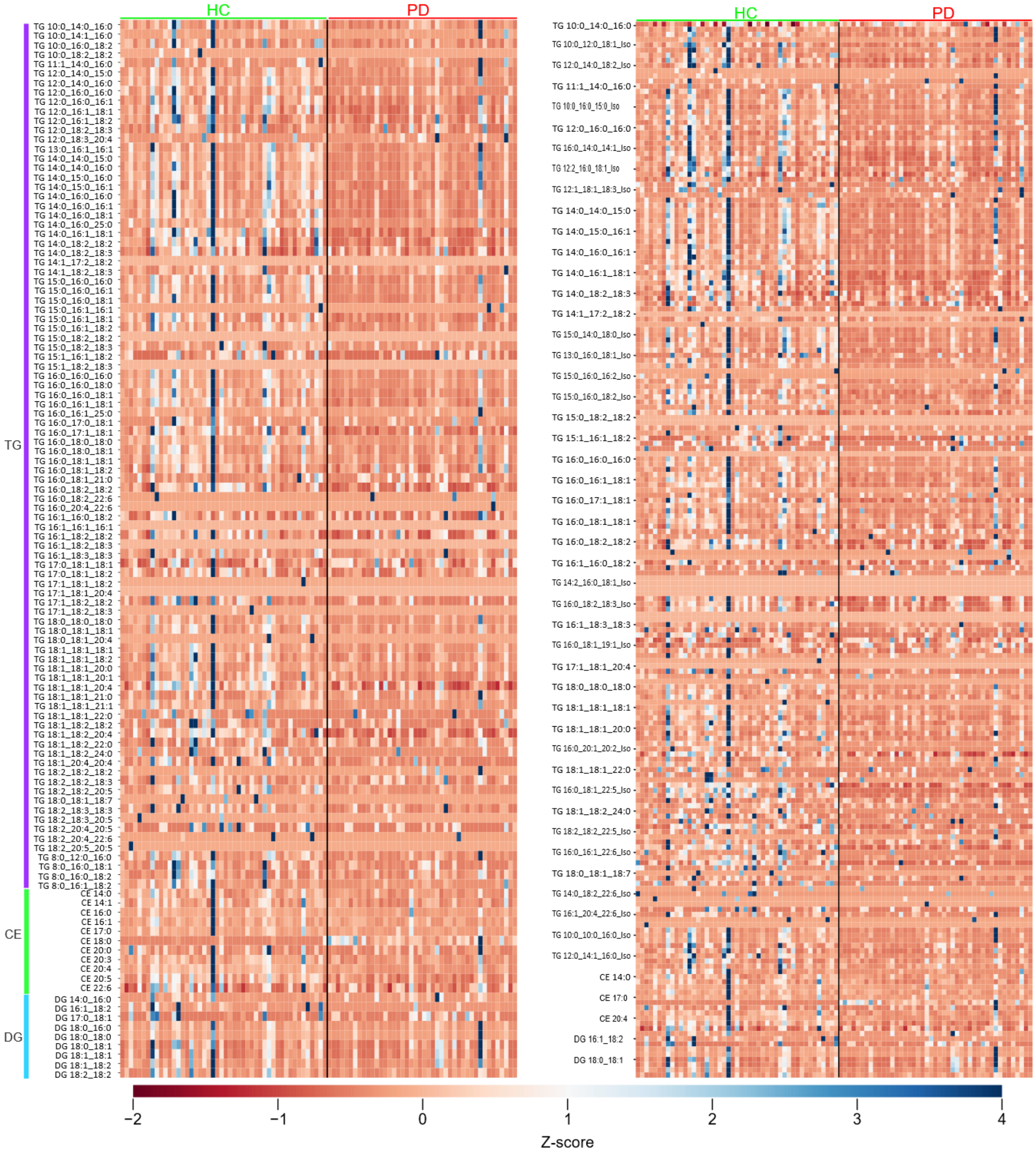

Supplementary Table 1: List of Internal standards (ISTD)[1]

Lipid Class	ISTD(s) per class
CE	TG 14:0_16:1_14:0 d5
DG	TG 14:0_16:1_14:0 d5
PC	PC 17:0/14:1 d5
	PC 17:0/18:1 d5
PC O-	PC O-18:1/18:1 d9
PE	PE 17:0/14:1 d5
	PE 17:0/18:1 d5
PG	PG 17:0/18:1 D5
PI	PI 17:0/14:1 d5
	PI 17:0/18:1 d5
PS	PS 17:0/14:1 d5
SM	SM d18:1/16:1 d9
	SM d18:1/18:1 d9
TG	TG 14:0_16:1_14:0 d5
	TG 17:0_17:1_17:0 d5
	TG 19:0_12:0_19:0 d5

[1] List of ISTD(s) per class spiked in the plasma/serum and the ones marked in violet used for quantification by normalizing the analyte to that ISTD

Supplementary Table 2: Limit of Detection (LOD and Limit of Quantification (LOQ) values[2]

Lipid Class	LOD (ng/mL)	LOQ (ng/mL)	Conc. Range (ng/mL)
**Cer**	**0.79**	**2.4**	**15 - 1920**
**FAHFA**	**0.31**	**0.97**	**9.38 - 1200**
**LPC**	**0.75**	**2.29**	**18.75 - 2400**
**LPE**	**1.51**	**4.57**	**5 - 640**
**PC**	**3.02**	**9.14**	**50 - 6400**
**PC O-**	**0.03**	**0.95**	**7.5 - 960**
**PE**	**0.68**	**2.07**	**7.5 - 960**
**PI**	**5.05**	**15.29**	**12.5 - 1600**
**PS**	**26.74**	**81.03**	**37.5 - 2400**
**SM**	**1.36**	**4.12**	**25 - 3200**

[2] LOD and LOQ values in ng/mL for each observed lipid class. LOD was found to be lower than the lowest point in the concentration range for each lipid class. The same was true for LOQ except for PI and PS lipid classes.

Supplementary Table 3. Phosphatidylcholine Standards purity[3]

PC	PLA2 digestion		SN model		Literature[1]		Literature[2]	
	SNA (%)	SN B (%)	SN A (%)	SN B (%)	SN A (%)	SN B (%)	SN A (%)	SN B (%)
**PC 16:0_18:1**	93.58 ± 7.78	6.42 ± 7.78	93.49 ± 1.72	6.51 ± 1.72	83.6	16.4	88	12
**PC 16:0_18:2**	97.53 ± 2.62	2.47 ± 2.62	94.70 ± 2.44	5.30 ± 2.44				
**PC 16:0_20:4**	99.48 ± 0.63	0.52 ± 0.63	98.19 ± 1.56	1.81 ± 1.56				
**PC 18:0_18:1**			95.49 ± 2.13	4.51 ± 2.13	90.7	9.3	96	4
**PC 18:0_18:2**	95.91 ± 5.78	4.09 ± 5.78	97.36 ± 1.28	2.64 ± 1.28				

Supplementary Table 4: Demographic data for participants of Parkinson's

[3] Abundance of the sn positional isomers (SNA and SN B) for commonly occurring glycerophosphocholine (PC) standards calculated using phospholipase A_2 (PLA_2) enzymatic activity, "SN regression" model and that reported in the literature.

cohort[4]

	Controls (HC)	Parkinson's patients (PD)
No. of Female participants	**17**	**16**
No. of Male participants	**28**	**31**
Average age (in years)	**62.0±9.9**	**62.0±13.6**

Supplementary Table 5: Significant features based on tSNE analysis[5]

MS1-based	MS2-based

[4] Average age and total umber of female and male participants across HC and PD group in Parkinson's cohort

[5] Significant features between the groups healthy control (HC) and Parkinson's disease (PD) resulting from MS1 based quantification in the first column and a corresponding co-eluting compositional isomer identified only based on prm-PASEF (on the right), also found to be significant between HC and PD.

Pre-dominant Lipid	**Co-eluting Isomer(s)**
PC 14:0_18:2	**PC 16:1_16:1**
PC 16:0_18:2	**PC 16:1_18:1**
PC 17:0_18:2	**PC 17:1_18:1**
PC 18:0_20:2	**PC 18:1_20:1**
PC 18:1_18:2	**PC 16:0_20:3**
PC 18:2_20:5	**PC 16:1_22:6**
PE 18:1_18:2	**PE 16:0_20:3**
TG 10:0_18:2_18:2	**TG 10:1_14:1_22:2**
TG 12:0_16:1_18:2	**TG 12:2_16:0_18:1 & TG 14:1_14:1_18:1**
TG 14:0_14:0_15:0	**TG 12:0_15:0_16:0**
TG 14:1_18:2_18:3	**TG 14:2_18:1_18:3**
TG 15:0_16:1_16:1	**TG 15:0_16:0_16:2 & TG 14:1_17:1_16:0 & TG 13:0_18:1_16:1**
TG 16:0_18:1_21:0	**TG 17:0_18:1_20:0**
TG 16:1_18:2_18:2	**TG 16:0_18:2_18:3 & TG 16:0_18:1_18:4**
TG 16:1_18:2_18:3	**TG 16:0_16:2_20:4**
TG 16:1_18:3_18:3	**TG 16:3_18:2_18:2 & TG 16:1_16:2_20:4**
TG 17:0_18:1_18:1	**TG 16:0_18:1_19:1**
TG 17:0_18:1_18:2	**TG 16:0_18:2_19:1**

Materials & Methods

We herewith confirm that the samples from Parkinson's cohort with the project number 837.311.12(842-F) used in this research complied with all relevant ethical regulations regarding the use of human study participants and was conducted in accordance to the criteria set by Rheinland-Pfalz.

Chemicals and Solvents

The following LC-MS grade solvents and reagents used in analytical workflow were purchased from Merck (Germany): water, methanol (MeOH), 2-propanol, formic acid (FA), triethylamine, ammonium formate, HPLC-grade methyl tert-butyl ether (MTBE). Sodium hydroxide used in the preparation of the recalibration solution was purchased from Thermo Fisher Scientific (Belgium). NIST plasma and serum SRMs were purchased from Merck (National Institute of Standards and Technology, USA). Phospholipase A2 from porcine pancreas (P6534) and Dulbecco's Phosphate Buffered Saline (D8662) used in the enzymatic digestion were purchased Merck (Darmstadt, Germany). $CaCl_2$ used in the enzymatic digestion was purchased from Sigma-Aldrich (Lot 87H0668).

Lipid Standards

Calibration Standards 1-hexadecanoyl-2-(9Z-octadecenoyl)-sn-glycero-3-phosphocholine (PC 16:0/18:1), 1-(9Z-octadecenoyl)-2-hexadecanoyl-sn-glycero-3-phosphocholine (PC 18:1/16:0), 1-hexadecanoyl-2-octadecadienoyl-sn-glycero-3 phosphocholine (PC 16:0/18:2), 1-hexadecanoyl-2-eicosatetraenoyl-sn-glycero-3-phosphocholine (PC 16:0/20:4), 1-octadecanoyl-2-(octadecadienoyl)-sn-glycero-3-phosphocholine (PC 18:0/18:2), 1,2-di-(octadecenoyl)-sn-glycero-3-phosphocholine (PC 18:1/18:1), 1-octadecanoyl-2-octadecenoyl-sn-glycero-3-phosphocholine (PC 18:0/18:1), 1-octadecenoyl-2-octadecanoyl-sn-glycero-3-phosphocholine (PC 18:1/18:0), 1-tetradecanoyl-2-octadecanoyl-sn-glycero-3-phosphocholine (PC 14:0/18:0), 1-octadecanoyl 2-tetradecanoyl-sn-glycero-3-phosphocholine (PC 18:0/14:0), 1-hexadecanoyl-2-octadecanoyl-sn-glycero-3-phosphocholine (PC 16:0/18:0), 1-octadecanoyl-2-hexadecanoyl-sn-glycero-3-phosphocholine (PC 18:0/16:0), 1-hexadecanoyl-2-(9Z-octadecenoyl)-sn-glycero-3-phosphoethanolamine (PE 16:0/18:1), 1-stearoyl-2-linoleoyl-sn-glycero-3-phosphoethanolamine (PE 18:0/18:2), 1-oleoyl-2-palmitoyl-sn-glycero-3-phosphoethanolamine (IsoPure) (PE 16:0/18:1(n9)), 1-oleoyl-2-palmitoyl-sn-glycero-3-phospho-L-serine (Isopure) (PS 16:0/18:1(n9)) were obtained from Merck (Avanti Polar Lipids, Inc., United States).

Internal Standards (ISTDs for quantification) 1-pentadecanoyl-2-oleoyl(d7)-sn-glycero-3-phosphate (PA-d7-15:0/18:1), 1-heptadecanoyl-2-(9Z-tetradecenoyl)-sn-glycero-3-phosphocholine (PC 17:0/14:1), 1-heptadecanoyl-2-(9Z-tetradecenoyl)-sn-glycero-3-phosphoethanolamine (PE 17:0/14:1), 1-heptadecanoyl-2-(9Z-tetradecenoyl)-sn-glycero-3-phosphoglycerol (PG 17:0/14:1), 1-heptadecanoyl-2-(9Z-tetradecenoyl)-sn-glycero-3-phosphoinositol (PI 17:0/14:1), 1-heptadecanoyl-2-(9Z-tetradecenoyl)-sn-glycero-3-phosphoserine (PS 17:0/14:1), N-(heptadecanoyl)-sphing-4-enine (Cer d18:1/17:0), cholest-5-en-3β-ol(d7) (cholesterol d7), 1-heptadecanoyl-glycero-3-phosphate (LPA 17:0), 1-(10Z-heptadecenoyl)-sn-glycero-3-phosphocholine (LPC 17:1), 1-(9Z-heptadecenoyl)-sn-glycero-3-phosphoethanolamine (LPE 17:1), 1-(9Z-heptadecenoyl)-glycero-3-phospho-(1'-sn-glycerol) (LPG 17:1), 1-(10Z-heptadecenoyl)-sn-glycero-3-phospho-(1'-myo-inositol) (LPI 17:1), 1-(9Z-heptadecenoyl)-glycero-3-phosphoserine (LPS 17:1), N-palmitoyl-D-erythro-sphingosine (d7) (Cer d18:1-d7/16:0), N-stearoyl-D-erythro-sphingosine (d7) (Cer d18:1-d7/18:0), N-lignoceroyl-D-erythro-sphingosine (d7) (Cer d18:1-d7/24:0), (N- hexadecenoyl -D-erythro-sphingosylphosphorylcholine-d9 ((SM d18:1/16:1)-d9), (N-oleoyl-D-erythro-sphingosylphosphorylcholine-d9 ((SM d18:1/18:1)-d9), (N-Nervonoyl-D-erythro-sphingosylphosphorylcholine-d9 ((SM d18:1/24:1)-d9), 5-[((13,13,14,14,15,15,16,16,16-d9)palmitoyl)hydroxy]-stearic acid (5-PAHSA-d9), 9-[((13,13,14,14,15,15,16,16,16-d9)palmitoyl)hydroxy]-stearic acid (9-PAHSA-d9), 1-heptadecanoyl-2-hydroxy-sn-glycero(d5)-3-phosphocholine (LPC 17:0 d5), 1-heptadecanoyl-2-myristoleoyl-sn-glycero(d5)-3-phosphocholine (PC 17:0/14:1 d5), 1-heptadecanoyl-2-oleoyl-sn-glycero(d5)-3-phosphocholine (PC 17:0/18:1 d5), 1-heptadecanoyl-2-eicosatrienoyl-sn-glycero(d5)-3-phosphocholine (PC 17:0/20:3 d5), 1-heptadecanoyl-2-hydroxy-sn-glycero(d5)-3-phosphoethanolamine (LPE 17:0 d5), 1-heptadecanoyl-2-myristoleoyl-sn-glycero(d5)-3-phosphoethanolamine (PE 17:0/14:1 d5), 1-heptadecanoyl-2-oleoyl-sn-glycero(d5)-3-ssphosphoethanolamine (PE 17:0/18:1 d5), 1-heptadecanoyl-2-myristoleoyl-sn-glycero(d5)-3-phosphoinositol (ammonium salt) (PI 17:0/14:1 d5), 1-heptadecanoyl-2-oleoyl-sn-glycero(d5)-3-phosphoinositol (ammonium salt) (PI 17:0/18:1 d5), 1-heptadecanoyl-2-docosatetraenoyl-sn-glycero(d5)-3-phosphoinositol (ammonium salt) (PI 17:0/22:4 d5), 1-heptadecanoyl-2-myristoleoyl-sn-glycero(d5)-3-phospho- L-serine (sodium salt) (PS 17:0/14:1 d5), 1-heptadecanoyl-2-oleoyl-sn-glycero(d5)-3-phospho- L-serine (sodium salt) (PS 17:0/18:1 d5), 1-heptadecanoyl-2-myristoleoyl-sn-glycero(d5)-3-phospho-(1'-rac-glycerol) (sodium salt) (PG 17:0/14:1 d5), 1-heptadecanoyl-

2-oleoyl-sn-glycero(d5)-3-phospho-(1'-rac-glycerol)(sodium salt) (PG 17:0/18:1 d5), 1,3(d5)-ditetradecanoyl-2-(9Z-hexadecenoyl)-glycerol (TG 14:0/16:1/14:0 d5), 1,3-diheptadecanoyl-2-(10Z-heptadecenoyl)-glycerol (d5) (TG 17:0/17:1/17:0 d5), and 1,3(d5)-dinonadecanoyl-2-dodecanoyl-glycerol (TG 19:0/12:0/19:0 d5) were also obtained from Merck (Avanti Polar Lipids, Inc., United States).

Sample Preparation

Liquid-Liquid Extraction for NIST plasma SRM: A liquid-liquid extraction (LLE) method based on MTBE/methanol (10:3; v/v) was used for the extraction of NIST plasma SRM. 20 µl NIST plasma SRM aliquots were used for the extraction and further extraction was carried out as per the protocol described in Lerner et al[3]. The same workflow was applied to a cohort consisting of 47 healthy individuals and 47 Parkinson's patients. To also evaluate the age and sex differences in Parkinson's disease, the cohort included 35 females and 59 males with ages ranging from 20 to 82 years old.

µL-flow Reversed phase Liquid chromatography

The LC separation method was the same as mentioned in Lerner et. al[3].

tims PASEF acquisition

prm-PASEF: The prm-PASEF experiments were performed using a TIMS-TOF pro instrument for the negative ion mode and TIMS-TOF flex operating in electrospray ionization mode for the positive ion mode (Bruker Daltonics, Germany). The basic MS parameters used were as described previously[3]. The target list used for prm-PASEF acquisition was initially generated in a CSV file format containing the following mandatory headers: Mass [*m/z* (Da)], Charge, Isolation Width [*m/z*], RT [s], RT Range [s], Start IM [1/K0], End IM [1/K0], CE [eV] and then imported to prm-PASEF method editor in compass Hystar. MS interval setting was set to 10s. The mass isolation width was set to 1Da whereas the RT range for each of the targets was to 60s and the mobility window set to 0.03 V.s/cm^2. A target list template has been shown in the **Supplementary Data 1**. After successfully importing the target list, the sampling rate for each of the targeted precursors is generated with the "Generate schedule" option. The scheduler plots in the prm-PASEF method editor get automatically updated consequently.

Retention time is one of the crucial parameters for precursor isolation in the prm-PASEF method. Thus, it becomes vital to have stringent control over the specified retention time. Although an RT range of 60 seconds is used to mitigate any potential RT shifts, an extreme RT shift might lead to partial isolation of the precursor and therefore an incomplete profiling for that precursor. The use of an even broader RT range can mitigate this issue, but it limits the number of precursors that can be targeted per scan cycle. An alternative approach reported to avoid this issue is using the "Live wrapping" option provided within the acquisition software used in this workflow. Here, we used a DDA acquisition run of a phospholipids control sample, covering all the major phospholipid classes, and one of the investigative samples at the beginning of the analytical sequence to monitor and infer RT, CCS, MS/MS, and m/z parameters of specific lipid species in these samples and align RT, when necessary, for subsequent prm-PASEF acquisition. Therefore, the RT Shift was evaluated by acquiring a mixture consisting of phospholipid standards (**Supplementary Data 9**) and/or one of the samples from the batch in a DDA acquisition mode. The RT in the prm-PASEF precursor target list was then updated manually and consequently, the target schedule had to be regenerated with every instance of RT shift.

Both the TIMS and mass calibration of the instrument was carried out on a weekly basis with the following peaks from the Agilent ESI LC-MS tuning mix [*m/z*, 1/K0: (322.0481, 0.7318 Vs cm−2), (622.0289, 0.9848 V.s/cm^2), (922.0097, 1.1895 V.s/cm^2), (1221,9906, 1.3820 V.s/cm^2)] in the positive mode, and [*m/z*, 1/K0: (666.01879, 1.0371 V.s/cm^2), (965.9996, 1.2255 V.s/cm^2), (1265.9809, 1.3785 V.s/cm^2)] in the negative mode. An online re-calibration of the data was performed immediately after each sample acquisition using a mixture of Agilent ESI LC-MS tune mix and 1 mM sodium formate (1:1) injected directly into the ESI source via a syringe pump. For this, the 20 min LC-MS run time was divided into three segments with the first segment (0.0 to 0.05 min) used for method equilibration, the second segment (0.05 min to 0.3 min) used for the injection of tune mix-sodium formate, and the last segment (0.3 min to 20 min) used for the sample data acquisition. The switch between the segments was achieved using a conventional 6-port divert valve. The peaks list from this mixture used for the recalibration is presented in **Supplementary Data 10**. Extensive formation of sodium adducts of lipids, particularly in positive ion mode, was detected when factory-recommended concentrations of sodium formate calibrant were used for online recalibration. Therefore, the concentration of sodium formate was reduced from 10 mM to 1 mM without compromising the performance.

Method Validation:

Limit of Detection (LOD) and Limit of Quantitation (LOQ) were calculated using one standard species for each lipid class following the bioanalytical method validation guidelines[4,5]. A linear concentration range consisting of 7 serially diluted points was used for each lipid class (**Supplementary Data 11**). The LOD and LLOQ were calculated using the following empirical relation:

$$LLOD = 3.3\frac{\sigma}{s}$$

$$LLOQ = 10\,\frac{\sigma}{s}$$

Where LLOD = Lower Limit of Detection

LLOQ = Lower Limit of Quantification

σ = Standard deviation of Response

s = slope of calibration curve

Linearity was calculated based on the following empirical relation:

$y = mx + c$

Where y= Normalised response of the standard to the class-specific ISTD

x= Normalised concentration of the standard to the class-specific ISTD

m= slope of the linear regression curve

c= Intercept of the linear regression curve.

To evaluate the linearity, the correlation coefficient (r^2) value was taken into consideration.

Data Processing

The samples acquired for RT Shift evaluation in DDA acquisition mode were processed using Metaboscape 2023b. The parameters used for this processing in metaboscape were as described in Lerner et al[3].

The samples acquired in prm-PASEF acquisition mode were processed mainly using Skyline v22.2[6]. The Skyline interface was set to molecule interface. The required transition list for processing in Skyline was generated externally using a csv format file. The transition list consisted of the following columns: molecule group, precursor name, precursor adduct, precursor *m/z*, precursor charge, product *m/z*, product charge, explicit retention time, explicit retention time window, explicit ion mobility, explicit ion mobility units. An additional column with the header "Note" can be added to provide a description for that transition row. A transition list template has been shown in **Supplementary Data 1**. The molecule setting was kept to the default configuration of the molecule interface in Skyline. The transition settings were kept as follow:

Prediction tab:

Precursor mass: Monoisotopic, Product ion mass: Monoisotopic, Collision energy: None, Declustering potential: None, Optimization library: None, Compensation Voltage: None, "Use optimization values when present" was left unchecked.

Filter:

Precursor adducts: [M+HCOOH-H], [M-H], Fragment adducts: [M+], Ion types: f, p, Precursor *m/z* exclusion window: 1 *m/z*, "Auto-select all matching transitions" option was kept checked.

Library:

No Library match was performed within skyline for prm acquired data.

Instrument:

Min *m/z*: 50, Max *m/z*: 1500, Dynamic min product *m/z* left unchecked, Method match tolerance *m/z* kept to 0.5.

Full-Scan:

MS1 Filtering

Isotopic peaks included: Count, Precursor mass analyzer: TOF, Peaks: 1, Resolving power: 20000, Isotope labeling enrichment: Default, Ignore SIM Scans left unchecked.

MS/MS Filtering:

Acquisition method: PRM, Product mass analyzer: TOF, Resolving Power: 20000, High Selectivity extraction kept checked, For Retention time filtering: All matching scans were included.

Ion-mobility:

No Ion mobility library was used. Window type kept to fixed and fixed width set to 0.03.

The Document setting was also kept to the default configuration of the molecule interface in Skyline.

The processing transition list is imported via Import → Transition List option. The samples are then imported via Import → Results option with Add single-injection replicates in files and no collision energy optimization being performed. The following settings were used for optimal visualization: Under View Menu, Arrange Graphs option set to Tiled, Transitions set to single, and Transform set to Savitzky-Golay smoothing. The automated peak picking performed during sample import for all the features was also always manually verified for the best peak picking of all individual features. After all the processing, a report is generated via Export → Report → Transition Results which consists of all the necessary information such as the analyte name, sample name, precursor and product m/z, RT, peak area, background, and peak rank for all the targets in all the imported samples.

Standard solutions for various models

For the evaluation of variation of sn2/sn1 fragment peak area ratio with the alkyl chain length in sn1, sn2 position and total amount of unsaturation in the lipid molecule, a phospholipid standard mix solution from major phospholipid classes such as PA, PC, PE, PG, PI, and PS was prepared at 0.5 µg/mL (each of the phospholipid standards used in this mix had an original stock solution of 1 mg/mL). The complete list of phospholipid standards used in this mix is shown in **Supplementary Data 2**.

For the "SN regression" model, commercially available standards of both the sn positional isomers (SNA and SNB) of PC 32:0, PC 34:0, PC 34:1, PC 36:1, PE 34:1, and PS 34:1 were used. A series of mixtures with both SN A and SN B isomers of all the aforementioned PC molecules were prepared in the ratio of 1:1, 1:2, 1:4, 1:8, 2:1, 4:1, and 8:1 (SNA:SNB). The sn2/sn1 ratio in each of the mixtures was calculated and a regression graph was generated. The regression coefficients obtained from this graph were used to estimate the SN isomeric ratio of various PC analytes in NIST plasma SRM.

Similarly, to estimate the proportion of isomers with different acyl chains from co-eluting precursors, standards of PC 36:2 namely PC 18:1/18:1 and PC 18:0/18:2, and PC 36:4 namely PC 16:0/20:4 and PC 18:/18:2 were used. A standard stock solution of 1 mg/mL was prepared for each of these standards. From the standard stock solution, standard mix solutions were prepared with each of these isomers in different proportions of 1:1, 2:1, 4:1, 8:1, and vice versa.

Calculation of the sn2/sn1 ratio

During processing via skyline, the product m/z in the transition list consisted of the carboxylate ion loss fragment of both the sn1 and sn2 acyl chain from the PC molecules. The resulting transition peak area from both the sn1 and sn2 acyl chains was then used to calculate the sn2/sn1 ratio corresponding to that lipid molecule(s).

For the evaluation of the ratio of sn positional isomers from co-eluting precursors, the sn2/sn1 fragment peak area ratio in the SNA:SNB mix of each of the aforementioned PC standards was calculated and then averaged to obtain a sn2/sn1 fragment peak area ratio which is thereby independent of the alkyl chain length and amount of unsaturation in the lipid molecule. This procedure was repeated for all the mixtures prepared for this model. A regression curve was obtained by plotting the sn2/sn1 fragment peak area ratio against the corresponding SNA:SNB ratio of the mixtures. For the sake of simplicity, the SNA:SNB ratio was converted to the proportion of SNA isomer in the whole mixture. The resulting curve is shown in **Figure 3b** (curve from PC SN ratio and isomer model merged sheet). The regression coefficient obtained from this curve was used to calculate the abundance of SNA and SNB in any mixture.

1)

$$SNA/(SNA + SNB) = \left(ln \left(\frac{\frac{sn2COO - fragment\ ion\ peak\ area}{sn1COO - fragment\ ion\ peak\ area}}{regression\ coefficient - 1} \right) \right) / (regression\ coefficient - 2)$$

where SNA and SNB are the amounts of SNA and SNB isomers respectively

regression coefficient 1&2 obtained by plotting the sn2/sn1 value against the corresponding SNA:SNB ratio

2)

$$(SNB) = 100 - (SNA)$$

where SNA and SNB are the amounts of SNA and SNB isomers respectively

Similarly, for the estimation of the proportion of conformational isomers with different acyl chains, the resulting transition peak area from both the sn1 and sn2 acyl chains is calculated and then summed up. This is performed for all the co-eluting isomeric precursors with their respective acyl chain fragments. To obtain the proportion of each co-eluting isomer, the summed fragment intensities of the isomer are normalized individually to the MS1 precursor peak area common to all the co-eluting isomeric structures. For instance, for coeluting isomers A and B with MS1 precursor peak area P, the fractions of A and B were calculated as:

1) $$Fraction\ of\ A = \left(\left(\frac{sn1+sn2\ fragment\ peak\ area\ of\ isomer\ A}{P}\right)\right)$$

2) $$Fraction\ of\ B = \left(\left(\frac{sn1 + sn2\ fragment\ peak\ area\ of\ isomer\ B}{P}\right)\right)$$

For TG and DG lipid class, the characteristic fragment ions $[M+NH4-(RCOOH+17)]^+$ arising from the loss of a free fatty acid(s) and ammonia from the precursor ion (M) was used for quantification. This fragment ion theoretically arises from each of the three fatty acyls at different sn position of the glycerol backbone. Usually, the most intense fragment ion out of the three characteristic fragment ion was used as the quantifier ion unless the most intense fragment ion turned out to be common between a co-eluting TG isomer.

PLA_2 Digestion

The enzyme used for site-specific enzymatic digestion of phospholipid was Phospholipase A_2. The enzyme is known to cleave specifically at the COO^- of the sn2 position of the glycerol backbone resulting in majorly the lyso species from the sn1 acyl chain of the glycerophospholipid. Two mixtures of PC standards were prepared with concentrations of 600 fmol (Mix A) and 300 fmol (Mix B) respectively. Both the mixtures were pipetted into 2mL Eppendorf tubes and dried under a stream of nitrogen gas. Dried lipid mixtures were resuspended in a mixture of 490 µl PBS buffer and 10 µL 100 mM $CaCl_2$. Mixtures were then subjected to a vortex for 2 min at 4 °C and 1050 rpm. After the vortex, 1 µL of the PLA_2 enzyme solution was added to both mixtures which were then incubated at 37 °C for 4 hours on a thermoblock. Following the digestion, the lipids were extracted using MTBE:Methanol protocol as described before.[3] The same procedure was repeated also for all PC standards individually.

Calculation of the Standard Purity after PLA_2 digestion

Validation of the SN prediction model was performed by comparing the purity of the PC standards predicted by the SN prediction model to the purity of the PC standards resulting from PLA_2 digestion. To calculate the purity of PC standards after digestion, the following relations were used:

1) $$Fraction\ of\ A = \left(\left(\frac{Peak\ area\ of\ SN1\ Lyso}{Peak\ area\ of\ SN1+Peak\ area\ of\ SN2\ lyso}\right)\right)$$

2) $$Fraction\ of\ B = \left(\left(\frac{Peak\ area\ of\ SN2\ Lyso}{Peak\ area\ of\ SN1+Peak\ area\ of\ SN2\ lyso}\right)\right)$$

Method applicability to Parkinson's disease

The same workflow was applied to a cohort consisting of 47 healthy individuals and 47 Parkinson's patients. To also evaluate the age and sex differences in the Parkinson's disease, the cohort included 35 females and 59 males with age ranging from 20 to

82 years old. Pathway enrichment analysis using the KEGG identifier of the significant lipids inParkinson's disease (PD) patient was performed using the Reactome's online analysis tool.

Software

The instrument calibrations and the data acquisition were controlled by Compass Hystar 6.3 and timsControl 5.1.6. Data processing was performed using Skyline 23.1. The processed data in skyline was exported via Export→Report option in a csv file format. Further processing such as quantification was performed in MS excel 2019. All the visualizations and statistics were produced using MS Office Professional Plus 2019, Python 3.10 (Spyder 5.1.5), R-studio(V), Inkscape (V5), Biorender.com, OpenAI 2024 and Origin 2021.

Statistics and Visualization

To perform differential expression analysis using Limma's linear modeling function (lmFit) followed by empirical Bayes moderation (eBayes)[7] which is more generic and more suitable for the differential evaluation of expression data. The robustness of the models (e.g., missing values) was tested by comparing the original Limma model of the group comparison against a model with imputed data (replacing zeros with a small close-to-zero number). However, in the original data, only a few data points were missing, and that also only in a few lipid features, the results show no differences in the two models and we decided against data imputation. An adjustment was made for the differential expression analysis for covariates including sex and age.

References:

(1) Wozny, K.; Lehmann, W. D.; Wozny, M.; Akbulut, B. S.; Brügger, B. A Method for the Quantitative Determination of Glycerophospholipid Regioisomers by UPLC-ESI-MS/MS. Anal Bioanal Chem 2019, 411 (4), 915–924. https://doi.org/10.1007/s00216-018-1517-5.

(2) Ekroos, K.; Ejsing, C. S.; Bahr, U.; Karas, M.; Simons, K.; Shevchenko, A. Charting Molecular Composition of Phosphatidylcholines by Fatty Acid Scanning and Ion Trap MS3 Fragmentation. J Lipid Res 2003, 44 (11), 2181–2192. https://doi.org/10.1194/jlr.D300020-JLR200.

(3) Lerner, R.; Baker, D.; Schwitter, C.; Neuhaus, S.; Hauptmann, T.; Post, J. M.; Kramer, S.; Bindila, L. Four-Dimensional Trapped Ion Mobility Spectrometry Lipidomics for High Throughput Clinical Profiling of Human Blood Samples. Nat Commun 2023, 14 (1). https://doi.org/10.1038/s41467-023-36520-1.

(4) INTERNATIONAL CONFERENCE ON HARMONISATION OF TECHNICAL REQUIREMENTS FOR REGISTRATION OF PHARMACEUTICALS FOR HUMAN USE ICH HARMONISED TRIPARTITE GUIDELINE VALIDATION OF ANALYTICAL PROCEDURES: TEXT AND METHODOLOGY Q2(R1).

(5) Shrivastava, A.; Gupta, V. Methods for the Determination of Limit of Detection and Limit of Quantitation of the Analytical Methods. Chronicles of Young Scientists 2011, 2 (1), 21. https://doi.org/10.4103/2229-5186.79345.

(6) Adams, K. J.; Pratt, B.; Bose, N.; Dubois, L. G.; St. John-Williams, L.; Perrott, K. M.; Ky, K.; Kapahi, P.; Sharma, V.; Maccoss, M. J.; Moseley, M. A.; Colton, C. A.; Maclean, B. X.; Schilling, B.; Thompson, J. W. Skyline for Small Molecules: A Unifying Software Package for Quantitative Metabolomics. J Proteome Res 2020, 19 (4), 1447–1458. https://doi.org/10.1021/acs.jproteome.9b00640.

(7) Ritchie, M. E.; Phipson, B.; Wu, D.; Hu, Y.; Law, C. W.; Shi, W.; Smyth, G. K. Limma Powers Differential Expression Analyses for RNA-Sequencing and Microarray Studies. Nucleic Acids Res 2015, 43 (7), e47. https://doi.org/10.1093/nar/gkv007.
