# Supplementary material for: prm-PASEF-Based Quantification and Isomeric Model for Extended Coverage of Human Plasma Lipidome in Parkinson’s Disease
Source: Anal Chem. 2025 Oct 27;97(44):24295–305. doi: 10.1021/acs.analchem.5c02340 (PMC12613151; doi:10.1021/acs.analchem.5c02340)
Supplement: Supplementary file 2 [file ac5c02340_si_002.pdf]

prm-PASEF-based quantification and isomeric model for extended coverage of human plasma lipidome in Parkinson's disease

Dhanwin Baker[a], Gabriel Gonzalez Escamilla[b], Daniel Janitschke[c], Yvan Devaux[d], Nils Schröter[e], Sergiu Groppa[f], Laura Bindila[g*]

Author

[a]Clinical[a] Lipidomics Unit, Institute of Physiological Chemistry, University Medical Center of the Johannes Gutenberg University Mainz, Duesbergweg 6, 55128, Mainz, Germany
E-mail: dhabaker@uni-mainz.de

[b]Gabriel Gonzalez Escamilla (GGE) - Department of Neurology, Universitätsklinikum des Saarlandes, Kirrberger Straße 100, 66421, Homburg, GermanyGermany
E-mail: ggonzale@uni-mainz.de

[c]Daniel Janitschke (DJ) - Department of Neurology, Universitätsklinikum des Saarlandes, Kirrberger Straße 100, 66421, Homburg, Germany
E-mail: daniel.janitschke@uks.eu

[d]Yvan Devaux (YD) - Cardiovascular Research Unit, Department of Precision Health, Luxembourg Institute of Health, 1 A-B Rue Thomas Edison, 1445 Strassen, Luxembourg
E-mail: yvan.devaux@lih.lu

[e]Nils Schröter (NS) – Clinic for Neurology and Neurophysiology, Universität Klinikum Freiburg, Breisacher Straße 64, 79106, Freiburg Germany
Email: nils.schroter@uniklinik-freiburg.de

[f]Sergiu Groppa (SG) – Department of Neurology, Universitätsklinikum des Saarlandes, Kirrberger Straße 100, 66421, Homburg, Germany
E-mail: sergiu.groppa@uks.eu

[a*]Laura Bindila (LB) - Clinical Lipidomics Unit, Institute of Physiological Chemistry, University Medical Center of the Johannes Gutenberg University Mainz, Duesbergweg 6, 55128, Mainz, Germany
*E-mail: bindila@uni-mainz.de Tel: +4961313925794; Fax: +4961313923536

Supplementary Data 1: Acquisition and processing transition list with all the lipid species targeted in negative and positive ion modes for the prm-PASEF method.

Supplementary Data 2: sn2/sn1 ratio from lipid species of major lipid classes obtained with both prm-PASEF and DDA-PASEF acquisition strategies.

Supplementary Data 3: Positive and negative descriptors for every lipid species such as RT, precursor m/z, product m/z, retention time window, and ion mobility (V.s/cm^2) used for data processing of prm-PASEF data. Quantified values of all lipid species in respective ionization modes, as mentioned in the article, in both MS1 and MS2-based workflow with the relative proportion of "sn positional" isomer and co-eluting compositional isomers exclusively indicated by suffix "_iso".

Supplementary Data 4: Calculation of LOD, LOQ (in picomolar (pM) concentration), and accuracy (in %) as shown in the article with a representative lipid standard from every lipid class.

Supplementary Data 5: Precursor target list with target lipid species and the corresponding descriptors used for the prm-PASEF method acquisition of glycerophospholipid mixture, also used as a QC for every experiment. The glycerophospholipid mixture consisted of a few lipid species from every lipid class.

Supplementary Data 6: "SN prediction model" for PC, PE, and PS lipid classes. Calculation of the isomeric abundance of "sn positional" isomer for various lipid species from PC, PE, and PS lipid classes in a standard mixture and NIST human plasma SRM; showcasing of matrix effect with calculation of abundance of isomerically pure commercially available internal standard PC 17:0/14:1 d5 in standard mixture, spiked in NIST plasma before and after extraction.

Supplementary Data 7: Calculation of the isomeric purity of PC standards in various concentration mixtures based on PLA_2 assay. Abundance of the sn positional isomers (SNA and SNB) for commonly occurring glycerophosphocholine (PC) standards calculated using phospholipase A_2 (PLA_2) enzymatic activity, "SN regression" model, and that reported in the literature.

Supplementary Data 8: Calculation of the isomeric abundance of PC compositional isomers in various concentration mixtures.

Supplementary Data 9: Quantification values of lipid species for all samples in Parkinson's cohort. Parkinson's cohort consists of two groups HC (healthy control) and PD (Parkinson's Disease) with 33 female and 59 male participants.

Supplementary Data 10: List of significant features arising from t-SNE analysis between healthy control (HC) and Parkinson's disease (PD) patients for both MS1 and MS2-based quantified values. The data shows the significant features for both unimputed (Original) and imputed data with missing values for certain lipid species. The data also shows the significant features when both age and sex are considered as variable for t-SNE analysis.
