# Supplementary material for: prm-PASEF-Based Quantification and Isomeric Model for Extended Coverage of Human Plasma Lipidome in Parkinson’s Disease
Source: Anal Chem. 2025 Oct 27;97(44):24295–305. doi: 10.1021/acs.analchem.5c02340 (PMC12613151; doi:10.1021/acs.analchem.5c02340)
Supplement: Supplementary file 13 [file ac5c02340_si_013.pdf]

Contents of Report

Created by https://lipidomicstandards.org, version v1.0.0

Separation Workflow 2
Overall study design 2
Lipid extraction 2
Analytical platform 2
Quality control 2
Method qualification and validation 3
Reporting 3

Sample Descriptions 3
NIST Human Plasma SRM 1950 / Human / Plasma 3

Lipid Class Descriptions 4
1) DG[M+NH4]+ / Lipid identification 4
1) DG[M+NH4]+ / Lipid quantification 4
2) TG[M+NH4]+ / Lipid identification 5
2) TG[M+NH4]+ / Lipid quantification 5
3) LPC[M+HCOO]- / Lipid identification 6
3) LPC[M+HCOO]- / Lipid quantification 6
4) LPC O[M+HCOO]- / Lipid identification 7
4) LPC O[M+HCOO]- / Lipid quantification 7
5) LPE[M-H]- / Lipid identification 8
5) LPE[M-H]- / Lipid quantification 8
6) LPE O[M-H]- / Lipid identification 9
6) LPE O[M-H]- / Lipid quantification 9
7) LPG[M-H]- / Lipid identification 10
7) LPG[M-H]- / Lipid quantification 10
8) LPI[M-H]- / Lipid identification 11
8) LPI[M-H]- / Lipid quantification 11
9) PC[M+HCOO]- / Lipid identification 12
9) PC[M+HCOO]- / Lipid quantification 12
10) PC O[M+HCOO]- / Lipid identification 13
10) PC O[M+HCOO]- / Lipid quantification 13
11) PE[M-H]- / Lipid identification 14
11) PE[M-H]- / Lipid quantification 14
12) PE O[M+HCOO]- / Lipid identification 15
12) PE O[M+HCOO]- / Lipid quantification 15
13) PG[M-H]- / Lipid identification 16
13) PG[M-H]- / Lipid quantification 16
14) PI[M-H]- / Lipid identification 17
14) PI[M-H]- / Lipid quantification 17
15) PS[M-H]- / Lipid identification 18
15) PS[M-H]- / Lipid quantification 18
16) SM[M+HCOO]- / Lipid identification 19
16) SM[M+HCOO]- / Lipid quantification 19
17) Cer[M+HCOO]- / Lipid identification 20
17) Cer[M+HCOO]- / Lipid quantification 20
18) FC[M+H-H2O]+ / Lipid identification 21
18) FC[M+H-H2O]+ / Lipid quantification 21
19) CE[M+NH4]+ / Lipid identification 22
19) CE[M+NH4]+ / Lipid quantification 22

Separation Workflow

Overall study design

Title of the study	prm-PASEF-based quantification and isomeric model for extended coverage of human plasma lipidome in Parkinson's disease		
Document creation date	12/12/2024	Corresponding Email	bindila@uni-mainz.de
Principle investigator	Dr. Laura Bindila	Is the workflow targeted or untargeted?	Targeted
Institution	Institute of Physiological Chemistry - Clinical Lipidomics Unit	Clinical	Yes

Lipid extraction

Extraction method	2-phase system	2-phase system	MTBE
pH adjustment	None	Were internal standards added prior extraction?	Yes

Analytical platform

Number of separation dimensions	One dimension	Ion source	ESI
Separation Type 1	LC	MS Level	MS1, MS2
Separation Mode 1	RP	Mass resolution for detected ion at MS1	High resolution
Separation window (1) for lipid analyte selection (±) in minutes	20	Resolution at m/z 200 at MS1	40000
RT verified by standard	No	Mass accuracy in ppm at MS1	3
CCS verified by standard	No	Mass window for precursor ion isolation (in Da total isolation window)	1
Separation of isobaric/isomeric interferece confirmed	Yes	Mass resolution for detected ion at MS2	Low resolution
Model for separation prediction	No	Resolution in Da at MS2	1
MS type	QTOF	Was/Were additional dimension/techniques used	No
MS vendor	Bruker		

Quality control

Blanks	No	Type of QC sample	Reference material
Quality control	Yes		

Method qualification and validation

Method validation	Yes	Precison	No
Lipid recovery	No	Accuracy	Yes
Dynamic quantification range	Yes	Guidelines followed	EMA
Limit of quantitation (LOQ)/Limit of detection (LOD)	Yes		

Reporting

Are reported raw data uploaded into repository?	Available on request	Additional comments	All data required to support the findings are provided as source data and supplementary material.
Raw data upload	Available on request		

Sample Descriptions

NIST Human Plasma SRM 1950 / Human / Plasma

Storage and collection conditions	Available	Additives	None
Provided preanalytical information	-	Were samples stored under inert gas?	No
Temperature handling original sample	4-8 °C	Additional preservation methods	No
Instant sample preparation	No	Biobank samples	No
Storage temperature	-80 °C		

Lipid Class Descriptions

1) DG[M+NH4]+ / Lipid identification

Lipid class	DG	Limit of detection	No
MS Level for identification	MS1, MS2	RT verified by standard	Yes
Identification level	Molecular species level	Separation of isobaric/isomeric interferece confirmed	Yes
Polarity mode	Positive	Model for separation prediction	Yes
Type of positive (precursor)ion	[M+NH4]+	Additional dimension/techniques	IMS
Fragments for identification		CCS verified by standard	Yes
Isotope correction at MS1	No	How was/were the additional dimension(s) used?	Annotation confidence and precursor isolation in method parameters
Isotope correction at MS2	No	Was a model used to predict lipid molecule separation?	No
MS1 verified by standard	Yes	Lipid Identification Software	Metaboscape, Skyline
MS2 verified by standard	Yes	Data manipulation	Smoothing
Background check at MS1	No	Nomenclature for intact lipid molecule	Yes
Background check at MS2	No	Nomenclature for fragment ions	Yes
Did you presume assumptions for identification?	No	Further identification remarks	-
Check on:	Isomeric overlap, Isobaric overlap, In-source fragmentation		

Fragments for identification:

Fragment name
-FA2(-H)-(H2O+NH3)
-FA1(-H)-(H2O+NH3)

1) DG[M+NH4]+ / Lipid quantification

Quantitative	Yes	Type I isotope correction	Yes
MS Level for quantification	MS1, MS2	Limit of quantification	S/N ratio > 10
Internal lipid standard(s) MS1		Normalization to reference	No
Internal lipid standard(s) MS2		Lipid Quantification Software	Homemade
Type of quantification	Internal standard amount	Batch correction	No
Response correction	No	Further quantification remarks	-

Internal lipid standard(s) MS1:

Internal standard	Endogenous subclass
TG(14:0/16:1/14:0)[D5]	

Internal lipid standard(s) MS2:

Internal standard	Fragment(s)	Endogenous subclass
TG(14:0/16:1/14:0)	-FA2(-H)-(H2O+NH3)	

2) TG[M+NH4]+ / Lipid identification

Lipid class	TG	Limit of detection	No
MS Level for identification	MS1, MS2	RT verified by standard	Yes
Identification level	Molecular species level	Separation of isobaric/isomeric interferece confirmed	Yes
Polarity mode	Positive	Model for separation prediction	Yes
Type of positive (precursor)ion	[M+NH4]+	Additional dimension/techniques	IMS
Fragments for identification		CCS verified by standard	Yes

Fragment name
-FA2(-H)-(H2O+NH3)
-FA1(-H)-(H2O+NH3)

Isotope correction at MS1	No	How was/were the additional dimension(s) used?	Annotation confidence and precursor isolation in method parameters
Isotope correction at MS2	No	Was a model used to predict lipid molecule separation?	No
MS1 verified by standard	Yes	Lipid Identification Software	Metaboscape, Skyline
MS2 verified by standard	Yes	Data manipulation	Smoothing
Background check at MS1	No	Nomenclature for intact lipid molecule	Yes
Background check at MS2	No	Nomenclature for fragment ions	Yes
Did you presume assumptions for identification?	No	Further identification remarks	-
Check on:	Isomeric overlap, Isobaric overlap, In-source fragmentation		

2) TG[M+NH4]+ / Lipid quantification

Quantitative	Yes	Type I isotope correction	Yes
MS Level for quantification	MS1, MS2	Limit of quantification	S/N ratio > 10
Internal lipid standard(s) MS1		Normalization to reference	No

Internal standard	Endogenous subclass
TG(14:0/16:1/14:0)[D5]	

Internal lipid standard(s) MS2		Lipid Quantification Software	Homemade

Internal standard	Fragment(s)	Endogenous subclass
TG(14:0/16:1/14:0)	-FA2(-H)-(H2O+NH3)	

Type of quantification	Internal standard amount	Batch correction	No
Response correction	No	Further quantification remarks	-

3) LPC[M+HCOO]- / Lipid identification

Lipid class	LPC	Limit of detection	No
MS Level for identification	MS1, MS2	RT verified by standard	Yes
Identification level	sn Position	Separation of isobaric/isomeric interferece confirmed	Yes
Polarity mode	Negative	Model for separation prediction	Yes
Type of negative (precursor)ion	[M+HCOO]-	Additional dimension/techniques	IMS
Fragments for identification	Fragment name: FA1(+O)	CCS verified by standard	Yes
Isotope correction at MS1	No	How was/were the additional dimension(s) used?	Annotation confidence and precursor isolation in method parameters
Isotope correction at MS2	No	Was a model used to predict lipid molecule separation?	No
MS1 verified by standard	Yes	Lipid Identification Software	Metaboscape, Skyline
MS2 verified by standard	Yes	Data manipulation	Smoothing
Background check at MS1	No	Nomenclature for intact lipid molecule	Yes
Background check at MS2	No	Nomenclature for fragment ions	Yes
Did you presume assumptions for identification?	No	Further identification remarks	-
Check on:	Isomeric overlap, Isobaric overlap, In-source fragmentation		

3) LPC[M+HCOO]- / Lipid quantification

Quantitative	Yes	Type I isotope correction	Yes
MS Level for quantification	MS1, MS2	Limit of quantification	S/N ratio > 10
Internal lipid standard(s) MS1	Internal standard: LPC 17:0/0:0[D5]; Endogenous subclass:	Normalization to reference	No
Internal lipid standard(s) MS2	Internal standard: LPC 17:0/0:0[D5]; Fragment(s): FA1(+O); Endogenous subclass:	Lipid Quantification Software	Homemade
Type of quantification	Internal standard amount	Batch correction	No
Response correction	No	Further quantification remarks	-

4) LPC O[M+HCOO]- / Lipid identification

Lipid class	LPC O	Limit of detection	No
MS Level for identification	MS1, MS2	RT verified by standard	Yes
Identification level	sn Position	Separation of isobaric/isomeric interferece confirmed	Yes
Polarity mode	Negative	Model for separation prediction	Yes
Type of negative (precursor)ion	[M+HCOO]-	Additional dimension/techniques	IMS
Fragments for identification		CCS verified by standard	Yes
Isotope correction at MS1	No	How was/were the additional dimension(s) used?	Annotation confidence and precursor isolation in method parameters
Isotope correction at MS2	No	Was a model used to predict lipid molecule separation?	No
MS1 verified by standard	Yes	Lipid Identification Software	Metaboscape, Skyline
MS2 verified by standard	Yes	Data manipulation	Smoothing
Background check at MS1	No	Nomenclature for intact lipid molecule	Yes
Background check at MS2	No	Nomenclature for fragment ions	Yes
Did you presume assumptions for identification?	No	Further identification remarks	-
Check on:	Isomeric overlap, Isobaric overlap, In-source fragmentation		

Fragments for identification:

Fragment name
1

4) LPC O[M+HCOO]- / Lipid quantification

Quantitative	Yes	Type I isotope correction	Yes
MS Level for quantification	MS1, MS2	Limit of quantification	S/N ratio > 10
Internal lipid standard(s) MS1		Normalization to reference	No
Internal lipid standard(s) MS2		Lipid Quantification Software	Homemade
Type of quantification	Internal standard amount	Batch correction	No
Response correction	No	Further quantification remarks	-

Internal lipid standard(s) MS1:

Internal standard	Endogenous subclass
LPC 17:0/0:0[D5]	

Internal lipid standard(s) MS2:

Internal standard	Fragment(s)	Endogenous subclass
LPC 17:0/0:0[D5]	FA1(+O)	

5) LPE[M-H]- / Lipid identification

Lipid class	LPE	Limit of detection	No
MS Level for identification	MS1, MS2	RT verified by standard	Yes
Identification level	sn Position	Separation of isobaric/isomeric interferece confirmed	Yes
Polarity mode	Negative	Model for separation prediction	Yes
Type of negative (precursor)ion	[M-H]-	Additional dimension/techniques	IMS
Fragments for identification	Fragment name: -FA1(-H)	CCS verified by standard	Yes
Isotope correction at MS1	No	How was/were the additional dimension(s) used?	Annotation confidence and precursor isolation in method parameters
Isotope correction at MS2	No	Was a model used to predict lipid molecule separation?	No
MS1 verified by standard	Yes	Lipid Identification Software	Metaboscape, Skyline
MS2 verified by standard	Yes	Data manipulation	Smoothing
Background check at MS1	No	Nomenclature for intact lipid molecule	Yes
Background check at MS2	No	Nomenclature for fragment ions	Yes
Did you presume assumptions for identification?	No	Further identification remarks	-
Check on:	Isomeric overlap, Isobaric overlap, In-source fragmentation		

5) LPE[M-H]- / Lipid quantification

Quantitative	Yes	Type I isotope correction	Yes
MS Level for quantification	MS1, MS2	Limit of quantification	S/N ratio > 10
Internal lipid standard(s) MS1	Internal standard: LPE 17:0/0:0[D5]; Endogenous subclass:	Normalization to reference	No
Internal lipid standard(s) MS2	Internal standard: LPE 17:0/0:0[D5]; Fragment(s): -FA1(-H); Endogenous subclass:	Lipid Quantification Software	Homemade
Type of quantification	Internal standard amount	Batch correction	No
Response correction	No	Further quantification remarks	-

6) LPE O[M-H]- / Lipid identification

Lipid class	LPE O	Limit of detection	No
MS Level for identification	MS1, MS2	RT verified by standard	Yes
Identification level	sn Position	Separation of isobaric/isomeric interferece confirmed	Yes
Polarity mode	Negative	Model for separation prediction	Yes
Type of negative (precursor)ion	[M-H]-	Additional dimension/techniques	IMS
Fragments for identification	Fragment name: -FA1(-H)	CCS verified by standard	Yes
Isotope correction at MS1	No	How was/were the additional dimension(s) used?	Annotation confidence and precursor isolation in method parameters
Isotope correction at MS2	No	Was a model used to predict lipid molecule separation?	No
MS1 verified by standard	Yes	Lipid Identification Software	Metaboscape, Skyline
MS2 verified by standard	Yes	Data manipulation	Smoothing
Background check at MS1	No	Nomenclature for intact lipid molecule	Yes
Background check at MS2	No	Nomenclature for fragment ions	Yes
Did you presume assumptions for identification?	No	Further identification remarks	-
Check on:	Isomeric overlap, Isobaric overlap, In-source fragmentation		

6) LPE O[M-H]- / Lipid quantification

Quantitative	Yes	Type I isotope correction	Yes
MS Level for quantification	MS1, MS2	Limit of quantification	S/N ratio > 10
Internal lipid standard(s) MS1		Normalization to reference	No
Internal lipid standard(s) MS2		Lipid Quantification Software	Homemade
Type of quantification	Internal standard amount	Batch correction	No
Response correction	No	Further quantification remarks	-

Internal lipid standard(s) MS1:

Internal standard	Endogenous subclass
LPE 17:0/0:0[D5]	

Internal lipid standard(s) MS2:

Internal standard	Fragment(s)	Endogenous subclass
LPE 17:0/0:0[D5]	-FA1(-H)	

7) LPG[M-H]- / Lipid identification

Lipid class	LPG	Limit of detection	No
MS Level for identification	MS1, MS2	RT verified by standard	Yes
Identification level	sn Position	Separation of isobaric/isomeric interferece confirmed	Yes
Polarity mode	Negative	Model for separation prediction	Yes
Type of negative (precursor)ion	[M-H]-	Additional dimension/techniques	IMS
Fragments for identification	Fragment name: -FA1(-H)	CCS verified by standard	Yes
Isotope correction at MS1	No	How was/were the additional dimension(s) used?	Annotation confidence and precursor isolation in method parameters
Isotope correction at MS2	No	Was a model used to predict lipid molecule separation?	No
MS1 verified by standard	Yes	Lipid Identification Software	Metaboscape, Skyline
MS2 verified by standard	Yes	Data manipulation	Smoothing
Background check at MS1	No	Nomenclature for intact lipid molecule	Yes
Background check at MS2	No	Nomenclature for fragment ions	Yes
Did you presume assumptions for identification?	No	Further identification remarks	-
Check on:	Isomeric overlap, Isobaric overlap, In-source fragmentation		

7) LPG[M-H]- / Lipid quantification

Quantitative	Yes	Type I isotope correction	Yes
MS Level for quantification	MS1, MS2	Limit of quantification	S/N ratio $>$ 10
Internal lipid standard(s) MS1	Internal standard: LPG 17:0/0:0[D5]; Endogenous subclass:	Normalization to reference	No
Internal lipid standard(s) MS2	Internal standard: LPG 17:0/0:0[D5]; Fragment(s): -FA1(-H); Endogenous subclass:	Lipid Quantification Software	Homemade
Type of quantification	Internal standard amount	Batch correction	No
Response correction	No	Further quantification remarks	-

8) LPI[M-H]- / Lipid identification

Lipid class	LPI	Limit of detection	No
MS Level for identification	MS1, MS2	RT verified by standard	Yes
Identification level	sn Position	Separation of isobaric/isomeric interferece confirmed	Yes
Polarity mode	Negative	Model for separation prediction	Yes
Type of negative (precursor)ion	[M-H]-	Additional dimension/techniques	IMS
Fragments for identification<br>**Fragment name**<br>-FA1(-H)		CCS verified by standard	Yes
Isotope correction at MS1	No	How was/were the additional dimension(s) used?	Annotation confidence and precursor isolation in method parameters
Isotope correction at MS2	No	Was a model used to predict lipid molecule separation?	No
MS1 verified by standard	Yes	Lipid Identification Software	Metaboscape, Skyline
MS2 verified by standard	Yes	Data manipulation	Smoothing
Background check at MS1	No	Nomenclature for intact lipid molecule	Yes
Background check at MS2	No	Nomenclature for fragment ions	Yes
Did you presume assumptions for identification?	No	Further identification remarks	-
Check on:	Isomeric overlap, Isobaric overlap, In-source fragmentation		

8) LPI[M-H]- / Lipid quantification

Quantitative	Yes	Type I isotope correction	Yes
MS Level for quantification	MS1, MS2	Limit of quantification	S/N ratio > 10
Internal lipid standard(s) MS1		Normalization to reference	No
Internal lipid standard(s) MS2		Lipid Quantification Software	Homemade
Type of quantification	Internal standard amount	Batch correction	No
Response correction	No	Further quantification remarks	-

Internal lipid standard(s) MS1:

Internal standard	Endogenous subclass
LPI 17:0/0:0[D5]	

Internal lipid standard(s) MS2:

Internal standard	Fragment(s)	Endogenous subclass
LPI 17:0/0:0[D5]	-FA1(-H)	

9) PC[M+HCOO]- / Lipid identification

Lipid class	PC	Limit of detection	No
MS Level for identification	MS1, MS2	RT verified by standard	Yes
Identification level	sn Position	Separation of isobaric/isomeric interferece confirmed	Yes
Polarity mode	Negative	Model for separation prediction	Yes
Type of negative (precursor)ion	[M+HCOO]-	Additional dimension/techniques	IMS
Fragments for identification		CCS verified by standard	Yes
Isotope correction at MS1	No	How was/were the additional dimension(s) used?	Annotation confidence and precursor isolation in method parameters
Isotope correction at MS2	No	Was a model used to predict lipid molecule separation?	No
MS1 verified by standard	Yes	Lipid Identification Software	Metaboscape, Skyline
MS2 verified by standard	Yes	Data manipulation	Smoothing
Background check at MS1	No	Nomenclature for intact lipid molecule	Yes
Background check at MS2	No	Nomenclature for fragment ions	Yes
Did you presume assumptions for identification?	No	Further identification remarks	-
Check on:	Isomeric overlap, Isobaric overlap, In-source fragmentation		

Fragments for identification:

Fragment name
-FA1(-H)-(CH3+HCOO)
-FA2(-H)-(CH3+HCOO)

9) PC[M+HCOO]- / Lipid quantification

Quantitative	Yes	Type I isotope correction	Yes
MS Level for quantification	MS1, MS2	Limit of quantification	S/N ratio > 10
Internal lipid standard(s) MS1		Normalization to reference	No
Internal lipid standard(s) MS2		Lipid Quantification Software	Homemade
Type of quantification	Internal standard amount	Batch correction	No
Response correction	No	Further quantification remarks	-

Internal lipid standard(s) MS1:

Internal standard	Endogenous subclass
PC(17:0/14:1)[D5]	

Internal lipid standard(s) MS2:

Internal standard	Fragment(s)	Endogenous subclass
PC(17:0/14:1)[D5]	-FA1(-H)-(CH3+HCOO)	
PC(17:0/14:1)[D5]	-FA2(-H)-(CH3+HCOO)	

10) PC O[M+HCOO]- / Lipid identification

Lipid class	PC O	Limit of detection	No
MS Level for identification	MS1, MS2	RT verified by standard	Yes
Identification level	sn Position	Separation of isobaric/isomeric interferece confirmed	Yes
Polarity mode	Negative	Model for separation prediction	Yes
Type of negative (precursor)ion	[M+HCOO]-	Additional dimension/techniques	IMS
Fragments for identification	Fragment name: -FA(-H)-(CH3+HCOO)	CCS verified by standard	Yes
Isotope correction at MS1	No	How was/were the additional dimension(s) used?	Annotation confidence and precursor isolation in method parameters
Isotope correction at MS2	No	Was a model used to predict lipid molecule separation?	No
MS1 verified by standard	Yes	Lipid Identification Software	Metaboscape, Skyline
MS2 verified by standard	Yes	Data manipulation	Smoothing
Background check at MS1	No	Nomenclature for intact lipid molecule	Yes
Background check at MS2	No	Nomenclature for fragment ions	Yes
Did you presume assumptions for identification?	No	Further identification remarks	-
Check on:	Isomeric overlap, Isobaric overlap, In-source fragmentation		

10) PC O[M+HCOO]- / Lipid quantification

Quantitative	Yes	Type I isotope correction	Yes
MS Level for quantification	MS1, MS2	Limit of quantification	S/N ratio > 10
Internal lipid standard(s) MS1	Internal standard: PC(O-18:1/18:1)[D9]; Endogenous subclass:	Normalization to reference	No
Internal lipid standard(s) MS2	Internal standard: PC(O-18:1/18:1)[D9]; Fragment(s): -FA(-H)-(CH3+HCOO); Endogenous subclass:	Lipid Quantification Software	Homemade
Type of quantification	Internal standard amount	Batch correction	No
Response correction	No	Further quantification remarks	-

11) PE[M-H]- / Lipid identification

Lipid class	PE	Limit of detection	No
MS Level for identification	MS1, MS2	RT verified by standard	Yes
Identification level	sn Position	Separation of isobaric/isomeric interferece confirmed	Yes
Polarity mode	Negative	Model for separation prediction	Yes
Type of negative (precursor)ion	[M-H]-	Additional dimension/techniques	IMS
Fragments for identification		CCS verified by standard	Yes

Fragment name
-FA2(-H)
-FA1(-H)

Isotope correction at MS1	No	How was/were the additional dimension(s) used?	Annotation confidence and precursor isolation in method parameters
Isotope correction at MS2	No	Was a model used to predict lipid molecule separation?	No
MS1 verified by standard	Yes	Lipid Identification Software	Metaboscape, Skyline
MS2 verified by standard	Yes	Data manipulation	Smoothing
Background check at MS1	No	Nomenclature for intact lipid molecule	Yes
Background check at MS2	No	Nomenclature for fragment ions	Yes
Did you presume assumptions for identification?	No	Further identification remarks	-
Check on:	Isomeric overlap, Isobaric overlap, In-source fragmentation		

11) PE[M-H]- / Lipid quantification

Quantitative	Yes	Type I isotope correction	Yes
MS Level for quantification	MS1, MS2	Limit of quantification	S/N ratio > 10
Internal lipid standard(s) MS1		Normalization to reference	No

Internal standard	Endogenous subclass
PE(17:0/14:1)[D5]	

Internal lipid standard(s) MS2		Lipid Quantification Software	Homemade

Internal standard	Fragment(s)	Endogenous subclass
PE(17:0/14:1)[D5]	-FA2(-H)	
PE(17:0/14:1)[D5]	-FA1(-H)	

Type of quantification	Internal standard amount	Batch correction	No
Response correction	No	Further quantification remarks	-

12) PE O[M+HCOO]- / Lipid identification

Lipid class	PE O	Limit of detection	No
MS Level for identification	MS1, MS2	RT verified by standard	Yes
Identification level	sn Position	Separation of isobaric/isomeric interferece confirmed	Yes
Polarity mode	Negative	Model for separation prediction	Yes
Type of negative (precursor)ion	[M+HCOO]-	Additional dimension/techniques	IMS
Fragments for identification	Fragment name: -FA2(-H)	CCS verified by standard	Yes
Isotope correction at MS1	No	How was/were the additional dimension(s) used?	Annotation confidence and precursor isolation in method parameters
Isotope correction at MS2	No	Was a model used to predict lipid molecule separation?	No
MS1 verified by standard	Yes	Lipid Identification Software	Metaboscape, Skyline
MS2 verified by standard	Yes	Data manipulation	Smoothing
Background check at MS1	No	Nomenclature for intact lipid molecule	Yes
Background check at MS2	No	Nomenclature for fragment ions	Yes
Did you presume assumptions for identification?	No	Further identification remarks	-
Check on:	Isomeric overlap, Isobaric overlap, In-source fragmentation		

12) PE O[M+HCOO]- / Lipid quantification

Quantitative	Yes	Type I isotope correction	Yes
MS Level for quantification	MS1, MS2	Limit of quantification	S/N ratio > 10
Internal lipid standard(s) MS1	Internal standard: PE(17:0/14:1)[D5]; Endogenous subclass:	Normalization to reference	No
Internal lipid standard(s) MS2	Internal standard: PE(17:0/14:1)[D5]; Fragment(s): -FA2(-H); Endogenous subclass:	Lipid Quantification Software	Homemade
Type of quantification	Internal standard amount	Batch correction	No
Response correction	No	Further quantification remarks	-

13) PG[M-H]- / Lipid identification

Lipid class	PG	Limit of detection	No
MS Level for identification	MS1, MS2	RT verified by standard	Yes
Identification level	sn Position	Separation of isobaric/isomeric interferece confirmed	Yes
Polarity mode	Negative	Model for separation prediction	Yes
Type of negative (precursor)ion	[M-H]-	Additional dimension/techniques	IMS
Fragments for identification		CCS verified by standard	Yes

Fragment name
-FA2(-H)
-FA1(-H)

Isotope correction at MS1	No	How was/were the additional dimension(s) used?	Annotation confidence and precursor isolation in method parameters
Isotope correction at MS2	No	Was a model used to predict lipid molecule separation?	No
MS1 verified by standard	Yes	Lipid Identification Software	Metaboscape, Skyline
MS2 verified by standard	Yes	Data manipulation	Smoothing
Background check at MS1	No	Nomenclature for intact lipid molecule	Yes
Background check at MS2	No	Nomenclature for fragment ions	Yes
Did you presume assumptions for identification?	No	Further identification remarks	-
Check on:	Isomeric overlap, Isobaric overlap, In-source fragmentation		

13) PG[M-H]- / Lipid quantification

Quantitative	Yes	Type I isotope correction	Yes
MS Level for quantification	MS1, MS2	Limit of quantification	S/N ratio > 10
Internal lipid standard(s) MS1		Normalization to reference	No

Internal standard	Endogenous subclass
PG(17:0/18:1)[D5]	

Internal lipid standard(s) MS2		Lipid Quantification Software	Homemade

Internal standard	Fragment(s)	Endogenous subclass
PG(17:0/18:1)[D5]	-FA2(-H)	
PG(17:0/18:1)[D5]	-FA1(-H)	

Type of quantification	Internal standard amount	Batch correction	No
Response correction	No	Further quantification remarks	-

14) PI[M-H]- / Lipid identification

Lipid class	PI	Limit of detection	No
MS Level for identification	MS1, MS2	RT verified by standard	Yes
Identification level	sn Position	Separation of isobaric/isomeric interferece confirmed	Yes
Polarity mode	Negative	Model for separation prediction	Yes
Type of negative (precursor)ion	[M-H]-	Additional dimension/techniques	IMS
Fragments for identification		CCS verified by standard	Yes

Fragment name
-FA2(-H)
-FA1(-H)

Isotope correction at MS1	No	How was/were the additional dimension(s) used?	Annotation confidence and precursor isolation in method parameters
Isotope correction at MS2	No	Was a model used to predict lipid molecule separation?	No
MS1 verified by standard	Yes	Lipid Identification Software	Metaboscape, Skyline
MS2 verified by standard	Yes	Data manipulation	Smoothing
Background check at MS1	No	Nomenclature for intact lipid molecule	Yes
Background check at MS2	No	Nomenclature for fragment ions	Yes
Did you presume assumptions for identification?	No	Further identification remarks	-
Check on:	Isomeric overlap, Isobaric overlap, In-source fragmentation		

14) PI[M-H]- / Lipid quantification

Quantitative	Yes	Type I isotope correction	Yes
MS Level for quantification	MS1, MS2	Limit of quantification	S/N ratio > 10
Internal lipid standard(s) MS1		Normalization to reference	No

Internal standard	Endogenous subclass
PI(17:0/14:1)[D5]	

Internal lipid standard(s) MS2		Lipid Quantification Software	Homemade

Internal standard	Fragment(s)	Endogenous subclass
PI(17:0/14:1)[D5]	-FA2(-H)	
PI(17:0/14:1)[D5]	-FA1(-H)	

Type of quantification	Internal standard amount	Batch correction	No
Response correction	No	Further quantification remarks	-

15) PS[M-H]- / Lipid identification

Lipid class	PS	Limit of detection	No
MS Level for identification	MS1, MS2	RT verified by standard	Yes
Identification level	sn Position	Separation of isobaric/isomeric interferece confirmed	Yes
Polarity mode	Negative	Model for separation prediction	Yes
Type of negative (precursor)ion	[M-H]-	Additional dimension/techniques	IMS
Fragments for identification		CCS verified by standard	Yes

Fragment name
-FA2(-H)
-FA1(-H)

Isotope correction at MS1	No	How was/were the additional dimension(s) used?	Annotation confidence and precursor isolation in method parameters
Isotope correction at MS2	No	Was a model used to predict lipid molecule separation?	No
MS1 verified by standard	Yes	Lipid Identification Software	Metaboscape, Skyline
MS2 verified by standard	Yes	Data manipulation	Smoothing
Background check at MS1	No	Nomenclature for intact lipid molecule	Yes
Background check at MS2	No	Nomenclature for fragment ions	Yes
Did you presume assumptions for identification?	No	Further identification remarks	-
Check on:	Isomeric overlap, Isobaric overlap, In-source fragmentation		

15) PS[M-H]- / Lipid quantification

Quantitative	Yes	Type I isotope correction	Yes
MS Level for quantification	MS1, MS2	Limit of quantification	S/N ratio > 10
Internal lipid standard(s) MS1		Normalization to reference	No

Internal standard	Endogenous subclass
PS(17:0/14:1)[D5]	

Internal lipid standard(s) MS2		Lipid Quantification Software	Homemade

Internal standard	Fragment(s)	Endogenous subclass
PS(17:0/14:1)[D5]	-FA2(-H)	
PS(17:0/14:1)[D5]	-FA1(-H)	

Type of quantification	Internal standard amount	Batch correction	No
Response correction	No	Further quantification remarks	-

16) SM[M+HCOO]- / Lipid identification

Lipid class	SM	Limit of detection	No
MS Level for identification	MS1, MS2	RT verified by standard	Yes
Identification level	sn Position	Separation of isobaric/isomeric interferece confirmed	Yes
Polarity mode	Negative	Model for separation prediction	Yes
Type of negative (precursor)ion	[M+HCOO]-	Additional dimension/techniques	IMS
Fragments for identification		CCS verified by standard	Yes

Fragment name
-(CH3+HCOO)

Isotope correction at MS1	No	How was/were the additional dimension(s) used?	Annotation confidence and precursor isolation in method parameters
Isotope correction at MS2	No	Was a model used to predict lipid molecule separation?	No
MS1 verified by standard	Yes	Lipid Identification Software	Metaboscape, Skyline
MS2 verified by standard	Yes	Data manipulation	Smoothing
Background check at MS1	No	Nomenclature for intact lipid molecule	Yes
Background check at MS2	No	Nomenclature for fragment ions	Yes
Did you presume assumptions for identification?	No	Further identification remarks	-
Check on:	Isomeric overlap, Isobaric overlap, In-source fragmentation		

16) SM[M+HCOO]- / Lipid quantification

Quantitative	Yes	Type I isotope correction	Yes
MS Level for quantification	MS1, MS2	Limit of quantification	S/N ratio > 10
Internal lipid standard(s) MS1		Normalization to reference	No

Internal standard	Endogenous subclass
SM(d18:1/16:1)[D9]	

Internal lipid standard(s) MS2		Lipid Quantification Software	Homemade

Internal standard	Fragment(s)	Endogenous subclass
SM(d18:1/16:1)[D9]-(CH3+HCOO)		

Type of quantification	Internal standard amount	Batch correction	No
Response correction	No	Further quantification remarks	-

17) Cer[M+HCOO]- / Lipid identification

Lipid class	Cer	Limit of detection	No
MS Level for identification	MS1, MS2	RT verified by standard	Yes
Identification level	sn Position	Separation of isobaric/isomeric interferece confirmed	Yes
Polarity mode	Negative	Model for separation prediction	Yes
Type of negative (precursor)ion	[M+HCOO]-	Additional dimension/techniques	IMS
Fragments for identification<br>**Fragment name**<br>FA1(-CH2O)		CCS verified by standard	Yes
Isotope correction at MS1	No	How was/were the additional dimension(s) used?	Annotation confidence and precursor isolation in method parameters
Isotope correction at MS2	No	Was a model used to predict lipid molecule separation?	No
MS1 verified by standard	Yes	Lipid Identification Software	Metaboscape, Skyline
MS2 verified by standard	Yes	Data manipulation	Smoothing
Background check at MS1	No	Nomenclature for intact lipid molecule	Yes
Background check at MS2	No	Nomenclature for fragment ions	Yes
Did you presume assumptions for identification?	No	Further identification remarks	-
Check on:	Isomeric overlap, Isobaric overlap, In-source fragmentation		

17) Cer[M+HCOO]- / Lipid quantification

Quantitative	Yes	Type I isotope correction	Yes
MS Level for quantification	MS1, MS2	Limit of quantification	S/N ratio > 10
Internal lipid standard(s) MS1<br>**Internal standard** / **Endogenous subclass**<br>Cer(d18:1/16:1)[D7] /		Normalization to reference	No
Internal lipid standard(s) MS2<br>**Internal standard** / **Fragment(s)** / **Endogenous subclass**<br>Cer(d18:1/16:1)[D7] / FA1(-CH2O) /		Lipid Quantification Software	Homemade
Type of quantification	Internal standard amount	Batch correction	No
Response correction	No	Further quantification remarks	-

18) FC[M+H-H2O]+ / Lipid identification

Lipid class	FC	RT verified by standard	Yes
MS Level for identification	MS1	Separation of isobaric/isomeric interferece confirmed	Yes
Identification level	Molecular species level	Model for separation prediction	Yes
Polarity mode	Positive	Additional dimension/techniques	IMS
Type of positive (precursor)ion	[M+H-H2O]+	CCS verified by standard	Yes
Isotope correction at MS1	No	How was/were the additional dimension(s) used?	Annotation confidence and precursor isolation in method parameters
MS1 verified by standard	Yes	Was a model used to predict lipid molecule separation?	No
Background check at MS1	No	Lipid Identification Software	Metaboscape, Skyline
Did you presume assumptions for identification?	No	Data manipulation	Smoothing
Check on:	Isomeric overlap, Isobaric overlap, In-source fragmentation	Nomenclature for intact lipid molecule	Yes
Limit of detection	No	Further identification remarks	-

18) FC[M+H-H2O]+ / Lipid quantification

Quantitative	Yes	Limit of quantification	S/N ratio > 10
MS Level for quantification	MS1	Normalization to reference	No
Internal lipid standard(s) MS1		Lipid Quantification Software	Homemade
Type of quantification	Internal standard amount	Batch correction	No
Response correction	No	Further quantification remarks	-
Type I isotope correction	Yes		

Internal standard	Endogenous subclass
Cholesterol[D7]	

19) CE[M+NH4]+ / Lipid identification

Lipid class	CE	Limit of detection	No
MS Level for identification	MS1, MS2	RT verified by standard	Yes
Identification level	Molecular species level	Separation of isobaric/isomeric interferece confirmed	Yes
Polarity mode	Positive	Model for separation prediction	Yes
Type of positive (precursor)ion	[M+NH4]+	Additional dimension/techniques	IMS
Fragments for identification	Fragment name: -NH3-FA	CCS verified by standard	Yes
Isotope correction at MS1	No	How was/were the additional dimension(s) used?	Annotation confidence and precursor isolation in method parameters
Isotope correction at MS2	No	Was a model used to predict lipid molecule separation?	No
MS1 verified by standard	Yes	Lipid Identification Software	Metaboscape, Skyline
MS2 verified by standard	Yes	Data manipulation	Smoothing
Background check at MS1	No	Nomenclature for intact lipid molecule	Yes
Background check at MS2	No	Nomenclature for fragment ions	Yes
Did you presume assumptions for identification?	No	Further identification remarks	-
Check on:	Isomeric overlap, Isobaric overlap, In-source fragmentation		

19) CE[M+NH4]+ / Lipid quantification

Quantitative	Yes	Type I isotope correction	Yes
MS Level for quantification	MS1, MS2	Limit of quantification	S/N ratio > 10
Internal lipid standard(s) MS1	Internal standard: TG(14:0/16:1/14:0)[D5]; Endogenous subclass:	Normalization to reference	No
Internal lipid standard(s) MS2	Internal standard: TG(14:0/16:1/14:0); Fragment(s): -FA2(-H)-(H2O+NH3); Endogenous subclass:	Lipid Quantification Software	Homemade
Type of quantification	Internal standard amount	Batch correction	No
Response correction	No	Further quantification remarks	-
